# Intra-islet insulin synthesis defects are associated with endoplasmic reticulum stress and loss of beta cell identity in human diabetes

**DOI:** 10.1007/s00125-022-05814-2

**Published:** 2022-10-25

**Authors:** Noemi Brusco, Guido Sebastiani, Gianfranco Di Giuseppe, Giada Licata, Giuseppina E. Grieco, Daniela Fignani, Laura Nigi, Caterina Formichi, Elena Aiello, Stefano Auddino, Giuseppe Quero, Chiara M. A. Cefalo, Francesca Cinti, Andrea Mari, Pietro M. Ferraro, Alfredo Pontecorvi, Sergio Alfieri, Andrea Giaccari, Francesco Dotta, Teresa Mezza

**Affiliations:** 1 Diabetes and Metabolic Disease Unit, Department of Medicine, Surgery and Neurosciences, University of Siena, Siena, Italy; 2 Fondazione Umberto Di Mario, c/o Toscana Life Sciences, Siena, Italy; 3 Endocrinologia e Diabetologia, Fondazione Policlinico Universitario Agostino Gemelli IRCCS, Roma, Italy; 4 Dipartimento di Medicina e Chirurgia Traslazionale, Università Cattolica del Sacro Cuore, Roma, Italy; 5 Pancreatic surgery unit, Pancreatic Advanced Research Center (CRMPG), Fondazione Policlinico Universitario Agostino Gemelli IRCCS, Roma, Italy; 6 Institute of Neuroscience, National Research Council, Padova, Italy; 7 U.O.S. Terapia Conservativa della Malattia Renale Cronica, Fondazione Policlinico Universitario A. Gemelli IRCCS, Roma, Italy; 8 Digestive Disease Center, Fondazione Policlinico Universitario Agostino Gemelli IRCCS, Roma, Italy

**Keywords:** Dedifferentiation, ER stress, Pancreatic islets, Proinsulin, Type 2 diabetes

## Abstract

**Aims/hypothesis:**

Endoplasmic reticulum (ER) stress and beta cell dedifferentiation both play leading roles in impaired insulin secretion in overt type 2 diabetes. Whether and how these factors are related in the natural history of the disease remains, however, unclear.

**Methods:**

In this study, we analysed pancreas biopsies from a cohort of metabolically characterised living donors to identify defects in in situ insulin synthesis and intra-islet expression of ER stress and beta cell phenotype markers.

**Results:**

We provide evidence that in situ altered insulin processing is closely connected to in vivo worsening of beta cell function. Further, activation of ER stress genes reflects the alteration of insulin processing in situ. Using a combination of 17 different markers, we characterised individual pancreatic islets from normal glucose tolerant, impaired glucose tolerant and type 2 diabetic participants and reconstructed disease progression.

**Conclusions/interpretation:**

Our study suggests that increased beta cell workload is accompanied by a progressive increase in ER stress with defects in insulin synthesis and loss of beta cell identity.

**Graphical abstract:**

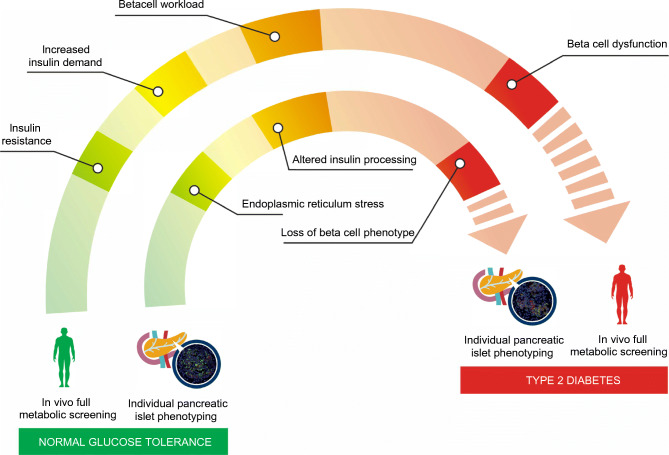

**Supplementary Information:**

The online version of this article (10.1007/s00125-022-05814-2) contains peer-reviewed but unedited supplementary material.

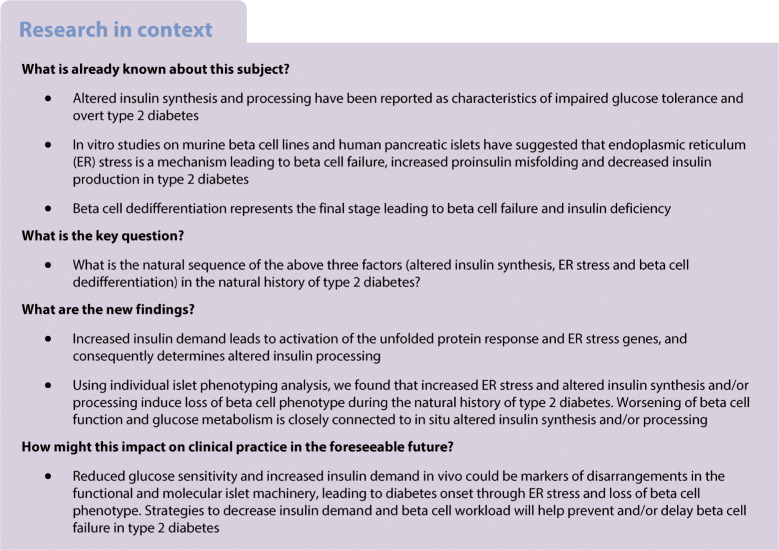



## Introduction

Beta cell dysfunction and consequent insulin deficiency contribute to the pathogenesis of type 2 diabetes [1]. Indeed, studies examining the timing and relationship between changes in beta cell molecular architecture, insulin secretion, insulin sensitivity and beta cell functional defects have identified the latter as the primary requisite for the development of hyperglycaemia [2–4].

Using a human model of partial pancreatectomy, we recently demonstrated that beta cell function and patterns of insulin secretion differed significantly among non-diabetic individuals, and that only pre-existing impairments in beta cell function, i.e. reduced first-phase insulin release (model-derived reduced glucose sensitivity and rate sensitivity) and defective proinsulin processing in the granules, predicted impairment in glucose tolerance and diabetes [5–7]. Thus, the actual determinant of diabetes development is the presence of a dysfunctional milieu, in which both morphological and functional alterations directly impact the beta cell secretory system [6, 8]. Hence, the prevailing opinion is that persistent metabolic stress (including insulin resistance) drives dysfunctional mature beta cells to phenotypically dedifferentiate or transdifferentiate into other islet endocrine cell types over time, and eventually to ‘beta cell exhaustion’ [6].

In vitro studies on murine beta cell lines and human pancreatic islets have identified endoplasmic reticulum (ER) stress as a mechanism leading to beta cell failure, increased proinsulin misfolding and decreased insulin production in type 2 diabetes [9]. In fact, ER protein overload is a key factor that could contribute to ER stress and subsequently to beta cell failure [10]. In particular, proinsulin biosynthesis, the primary driver of ER protein load in beta cells, can increase up to 50-fold in response to insulin resistance [11]. The increased rate of proinsulin synthesis, together with alterations in the ER environment, can cause accumulation of misfolded proinsulin. Consequently, increased proinsulin synthesis stimulates the ‘adaptive UPR’ (unfolded protein response) in order to re-establish ER homeostasis [12] which, if not resolved, may lead to terminal UPR and ER stress.

In this study, we finally linked full in vivo metabolic profiles to in situ molecular analyses of pancreatic islets of living human donors. We were able to reconstruct the putative alterations and the molecular mechanisms occurring in pancreatic islets during the natural history of type 2 diabetes by analysing pancreatic tissue samples obtained from fully metabolically characterised individuals classified into normal glucose tolerant (NGT), impaired glucose tolerant (IGT) and type 2 diabetic participants. We analysed proinsulin and insulin staining pattern variables and gene expression profiles at individual islet level with clinical/metabolic data from the same individuals, focusing on insulin processing, ER stress and loss of beta cell phenotype.

## Methods

### Participants, metabolic screening and surgical procedures

Eighteen patients (12 female; six male; mean age 65.1 ± 2.23 [years ± SEM]) undergoing pylorus-preserving pancreatoduodenectomy were recruited from January 2017 to July 2019 at the Digestive Surgery Unit and studied at the Centre for Endocrine and Metabolic Diseases unit (Agostino Gemelli University Hospital, Rome, Italy). All underwent complete metabolic screening including OGTT and mixed meal test (MMT).

The study protocol (ClinicalTrials.gov registration no. NCT02175459) was approved by the local ethics committee (P/656/CE2010 and 22573/14) (Rome, Italy) and all participants provided written informed consent, which was followed by a comprehensive medical evaluation.

**Fig. 1:**
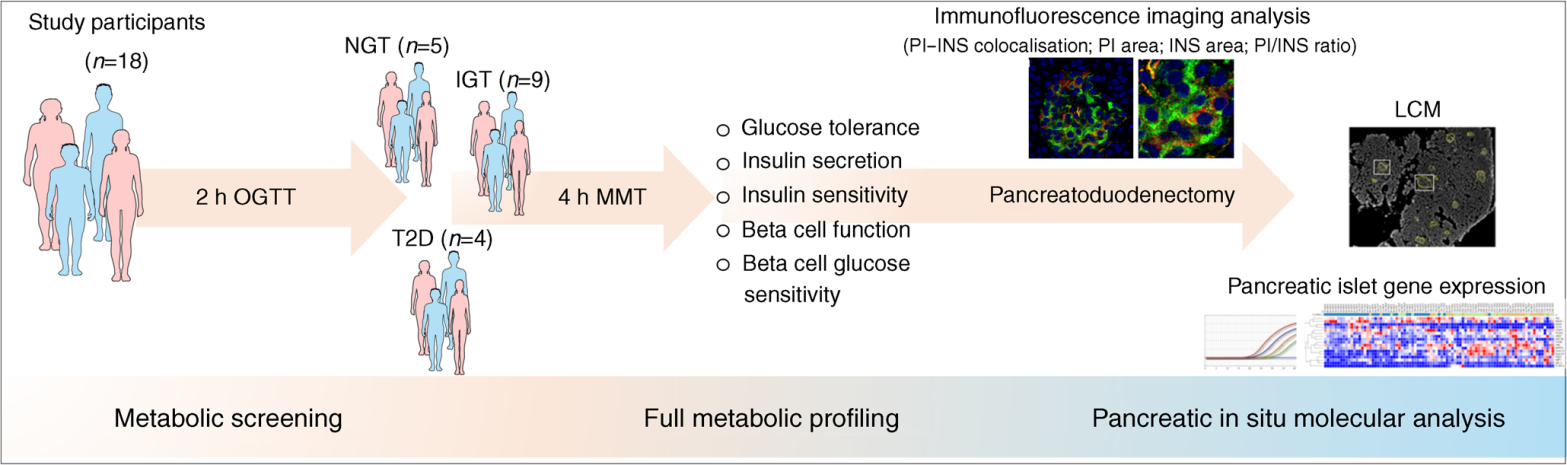
Study design and metabolic characterisation of NGT, IGT and type 2 diabetic participants. Study workflow scheme starting from individual metabolic profiling to molecular analysis of pancreatic islets in pancreas biopsies. INS, insulin; PI, proinsulin; T2D, type 2 diabetic. Graphics designed using Biomedical PowerPoint Toolkit from Motifolio (https://www.motifolio.com/)

Participants were metabolically profiled before undergoing surgery (Fig. 1). Based on the thresholds set by the ADA for fasting glucose, HbA_1c_ and 2 h glucose level during an OGTT in the days immediately before surgery, participants were classified as NGT (*n*=5), IGT (*n*=9) or with disease onset longer than 1 year (*n*=4). All 18 participants underwent both an OGTT and an MMT to evaluate insulin secretion (from C-peptide deconvolution) [7] (Table 1).

**Table 1:** Clinical and metabolic characteristics of participants

Clinical variable	NGT (*n*=5)	IGT (*n*=9)	Type 2 diabetes (*n*=4)	*p* value
Age	67.2 ± 19.9	67.1 ± 10.2	69.7 ± 5.43	0.90
Sex (M:F)	2:3	3:6	1:3	
BMI (kg/m^2^)	26.3 ± 2.83	25.3 ± 3.63	24.7 ± 4.46	0.88
HbA_1c_ (mmol/mol)	40.2 ± 4.97	40.5 ± 2.08	48.0 ± 9.17	0.35
HbA_1c_ (%)	5.82 ± 0.49	5.80 ± 0.16	6.58 ± 0.79	0.24
Total cholesterol (mmol/l)	4.25 ± 0.16	5.18 ± 1.30	4.89 ± 1.27	0.37
HDL-cholesterol (mmol/l)	1.01 ± 0.34	1.28 ± 0.34	1.05 ± 0.46	0.38
LDL-cholesterol (mmol/l)	3.0 ± 1.35	3.47 ± 0.91	2.95 ± 1.07	0.74
Triglycerides (mmol/l)	1.39 ± 0.49	1.54 ± 0.66	1.97 ± 0.77	0.13
Fasting glucose (mmol/l)	4.77 ± 0.99	5.1 ± 0.47	8.3 ± 2.95	0.02
Fasting insulin (pmol/l)	39.2 ± 15.1	77.0 ± 45.6	127.7 ± 77.7	0.02
Fasting C-peptide (ng/ml)	1.48 ± 0.76	1.67 ± 0.72	2.13 ± 1.65	0.13
Proinsulin/insulin ratio	0.28 ± 0.16	0.32 ± 0.06	1.28 ± 0.50	<0.01
Diabetes duration	–	–	4.00 ± 3.93	

Data are presented as means ± SD

Living donors were classified according to their glucose tolerance before surgery into NGT (*n*=5), IGT (*n*=9) and type 2 diabetes (*n*=4)

*p*<0.05 is considered statistically significant

M:F, male:female

Fasting glucose (*p*=0.05) and mean glucose at OGTT were both significantly increased only in participants with diabetes (*p*<0.01 for all time-points; see electronic supplementary material [ESM] Fig. 1a), as expected on the basis of the classification. Moreover, fasting insulin levels were significantly increased in IGT and type 2 diabetes groups (*p*=0.05), while mean insulin and C-peptide levels during OGTT significantly increased over time only in the IGT group (respectively, ESM Fig. 1b, *p*<0.05 for times 60 min, 90 min and 120 min; and ESM Fig. 1c, *p*<0.05 for times 60 min and 120 min). For detailed methods regarding metabolic screening and surgical procedures please refer to the ESM Methods section.

### Proinsulin–insulin immunofluorescence of human pancreatic sections and imaging analysis

Frozen pancreatic tissue sections were analysed through double immunofluorescence to evaluate the expression patterns of proinsulin and insulin. Primary antibodies Polyclonal Guinea Pig Anti-Human Insulin (cat. A0564, Agilent Technologies, Santa Clara, CA, USA) diluted 1:2000 and Mouse Monoclonal Anti-Human Proinsulin (cat. GS9A8–Developmental Study Hybridoma Bank, Iowa City, IA, USA) (epitope: B–C junction of proinsulin spanning aa 26–37) [13–15] diluted 1:100 in PBS 1X supplemented with 1% BSA overnight in a damp chamber at 4°C were used. Images were acquired using a Leica TCS SP5 confocal laser scanning microscope system (Leica Microsystems, Wetzlar, Germany) and analysed using Volocity 6.3 software (Perkin Elmer, Waltham, MA, USA). For detailed methods please refer to the ESM Methods section.

### Laser capture microdissection of human pancreatic islets

Pancreatic human tissue samples from *n*=5 NGT, *n*=9 IGT and *n*=4 type 2 diabetic living donors (Table 1) were frozen in Tissue-Tek OCT compound (Sakura Finetek Europe, the Netherlands) and then 7-μm-thick sections were cut from frozen OCT blocks. Laser capture microdissection (LCM) was performed using the Arcturus XT Laser-Capture Microdissection system (Arcturus Engineering, Mountain View, CA, USA). Human pancreatic islets were subsequently visualised through beta cell autofluorescence and captured using CapSure™ HS LCM Caps (ThermoFisher Scientific, Waltham, MA, USA) and infrared (IR) laser. For detailed methods please refer to the ESM Methods section.

### Gene expression analysis of pooled and individual human pancreatic islets

Total RNA was extracted from each LCM sample using PicoPure RNA Isolation Kit Arcturus (cat. kit0204, ThermoFisher Scientific) following the manufacturer’s procedure. For gene expression analysis, a reverse transcriptase reaction was performed using SuperScript VILO cDNA Synthesis Kit (cat. 11754050, ThermoFisher Scientific) and then pre-amplified. Real-time PCR analysis was performed using the TaqMan gene expression assays (ThermoFisher Scientific) reported in ESM Table 1. For pooled islets, RNA integrity (RIN) and concentration were checked using capillary electrophoresis with a 2100 Bioanalyzer with RNA Pico Chips (cat. 5067-1513, Agilent Technologies, Santa Clara, CA, USA). We excluded RNA samples with RIN<5.0 (ESM Table 2).

Individual pancreatic islets were characterised by analysing two consecutive frozen pancreatic tissue sections from each participant. The LCM isolation procedure was performed as described above, isolating one individual islet at a time. RNA extraction and gene expression analysis were performed as reported above. Specificity of the individual captured islet was checked by evaluating *AMY2A* (encoding the exocrine marker amylase 2A) mRNA expression (ESM Table 3). For detailed methods please refer to the ESM Methods section.

### Statistical analysis

#### Statistics for clinical data

Continuous variables were summarised as mean ± SEM and categorical variables as frequencies and percentages, unless otherwise indicated. Normality of distribution was assessed by generation of histograms and quantile–quantile plots. Since samples did not deviate significantly from normal, differences in means across groups at baseline were tested by ANOVA. The relationship between variables was derived by linear regression analysis. For measurement of glucose, insulin and C-peptide, we evaluated third-level interactions by including a product term of time × glucose tolerance in the model. A two-tailed *p* value <0.05 was considered statistically significant. Analyses were performed using Stata 15.1 (Stata Corp, TX, USA).

#### Statistics for in situ molecular data

Imaging analysis results and real-time PCR data were expressed as mean ± SD. Statistical analyses were performed using GraphPad Prism 9 software (San Diego, CA, USA). Comparisons between two groups were carried out using Mann–Whitney *U* test (for non-normally distributed data) or Wilcoxon matched signed rank test. Multiple comparisons were performed using ordinary one-way ANOVA and Kruskal–Wallis test followed by Tukey’s or Dunn’s post hoc test. The correlation analysis was performed by Spearman’s non-parametric test. Differences were considered as statistically significant with *p* values less than 0.05.

Correlation matrix analysis of multiple parameters was conducted using Spearman correlation analysis to evaluate the association between pooled LCM islets gene expression values, immunofluorescence imaging parameters and clinical data. *r* values and confidence intervals were taken into consideration to evaluate the impact of the correlation. Hierarchical clustering analysis, dendrogram generation and *k*-means clustering analyses were performed using Morpheus (https://software.broadinstitute.org/morpheus). Principal Components Analysis (PCA) and Eigenvector analysis were performed using GraphPad Prism 9 software.

## Results

### In situ proinsulin synthesis is altered in pancreatic beta cells of IGT and type 2 diabetic participants

**Fig. 2:**
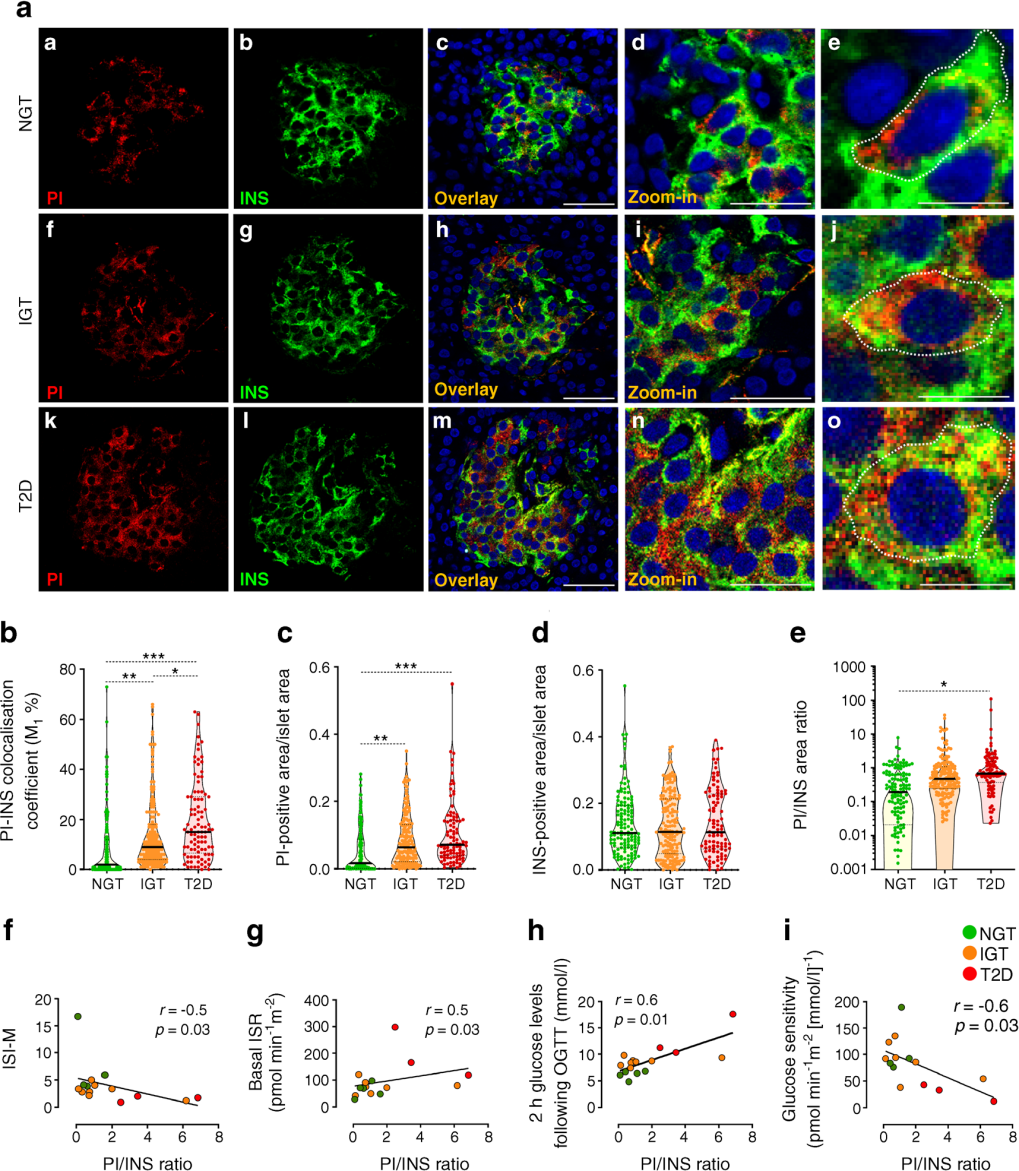
In situ proinsulin/insulin ratio and colocalisation rate are altered in pancreatic islets of IGT and type 2 diabetic participants. (**a**) Double immunofluorescence images showing the expression of PI (red) (panels a, f, k), INS (green) (panels b, g, l), DAPI (nuclei, blue) and overlay channels (yellow) (panels c, h, m) in frozen pancreatic tissue sections from NGT, IGT and type 2 diabetic participants. Scale bars, 50 μm. Digital zoom-in overlay images are reported in panels d, i and n; scale bars, 30 μm. High-resolution images of single beta cells of NGT, IGT and type 2 diabetic participants are reported in panels e, j and o; scale bars, 10 μm. Violin plot graphs showing PI–INS colocalisation rate (%) (**b**), PI-positive area (**c**), INS-positive area (**d**) and PI/INS area ratio (plotted on log_10_ axis) (**e**), measured in pancreatic islets (NGT *n*=131; IGT *n*=168; type 2 diabetes *n*=97) of *n*=5 NGT, *n*=9 IGT and *n*=5 type 2 diabetic participants. *p<0.05, **p<0.01, ***p<0.001, one-way ANOVA and Tukey’s multiple comparisons post hoc test. Correlation analysis of in situ PI/INS area ratio with ISI-M (*r*=−0.5, *p*=0.03) (**f**), basal ISR (*r*=0.5, *p*=0.03) (**g**), 2 h glucose levels at OGTT (*r*=0.6, *p*=0.03) (**h**) and glucose sensitivity (*r*=−0.6, *p*=0.03) (**i**). Correlations included all participants with available metabolic/clinical measures. *p* and *r* values were obtained using Spearman correlation test. INS, insulin; PI, proinsulin; T2D, type 2 diabetic

To evaluate in situ alteration of insulin synthesis and/or processing, we explored the expression and distribution of insulin and of its precursor proinsulin in frozen pancreatic tissue sections obtained from pancreas biopsies collected during surgery from NGT, IGT and type 2 diabetic participants (Table 1). Multiplex immunofluorescence staining and imaging analysis (Fig. 2a) were performed using two previously validated antibodies specifically recognising insulin and proinsulin without any reported cross-detection, as previously shown [13–15].

We measured proinsulin and insulin in pancreatic tissue sections of NGT, IGT and type 2 diabetic participants. Overall, we analysed 131 islets from NGT participants, 168 islets from IGT participants and 97 islets from type 2 diabetic participants.

In pancreatic beta cells of NGT participants, proinsulin–insulin double immunofluorescence analysis showed distinct signals of proinsulin and insulin (Fig. 2a, panels a–e), in line with other studies [15]. A z-stack 3D deconvolution analysis further confirmed the observed distribution (ESM Fig. 2a). Intracellular high-resolution images (Fig. 2a, panel e) and 2D graph analysis (ESM Fig. 3) showed that insulin is more widely distributed than proinsulin in beta cells of NGT islets.

In pancreatic beta cells of IGT participants, proinsulin was partially colocalised with insulin (Fig. 2a, panels f–j; ESM Fig. 2b), while in type 2 diabetic beta cells the majority of proinsulin signal colocalised with insulin (Fig. 2a, panels k–o; ESM Fig. 2c). In fact, proinsulin–insulin colocalisation rate gradually increased from NGT to IGT to type 2 diabetes (*p*<0.0001) (NGT: 8.7%; IGT: 14.1%; type 2 diabetes: 21.5% [colocalisation rate mean values]) (Fig. 2b).

In situ proinsulin and insulin imaging quantification analysis (Fig. 2b,e; ESM Fig. 4) showed that proinsulin area progressively increased from NGT to type 2 diabetes (*p*<0.0001) (NGT=0.054; IGT=0.085; type 2 diabetes=0.117 [proinsulin area/islet area mean values]) (Fig. 2c; ESM Figs 3, 4), while insulin area did not significantly differ among the three groups (Fig. 2d; ESM Figs 3, 4). Finally, we observed that proinsulin/insulin area ratio progressively increased in pancreatic islets from NGT to type 2 diabetes (*p*=0.03) (NGT=0.5; IGT=1.6; type 2 diabetes=3.3 [proinsulin/insulin area ratio mean values]) (Fig. 2e).

Overall, these data highlight an increased expression of proinsulin and altered proinsulin–insulin intracellular colocalisation in beta cells in human pancreatic samples from IGT and type 2 diabetic participants.

### In vivo beta cell dysfunction is linked to changes in insulin synthesis in situ

Correlation analysis between proinsulin–insulin imaging variables and clinical/metabolic outcomes showed that in situ proinsulin/insulin ratio was inversely correlated with Matsuda insulin sensitivity index [16] (ISI-M) (*r*=−0.5, *p*=0.03) (Fig. 2f), and positively correlated with basal insulin secretion rate (ISR) (*r*=0.5, *p*=0.03) (Fig. 2g) and 2 h glucose levels at OGTT (*r*=0.6, *p*=0.01) (Fig. 2h). Of note, we observed that increased proinsulin/insulin ratio in beta cells was associated with a reduced in vivo beta cell glucose sensitivity (*r*=−0.6, *p*=0.03) (Fig. 2i). Collectively, these results indicate altered insulin processing, which, in turn, reflects increased insulin demand and in vivo beta cell dysfunction.

### ER gene changes are associated with altered insulin processing in situ

**Fig. 3:**
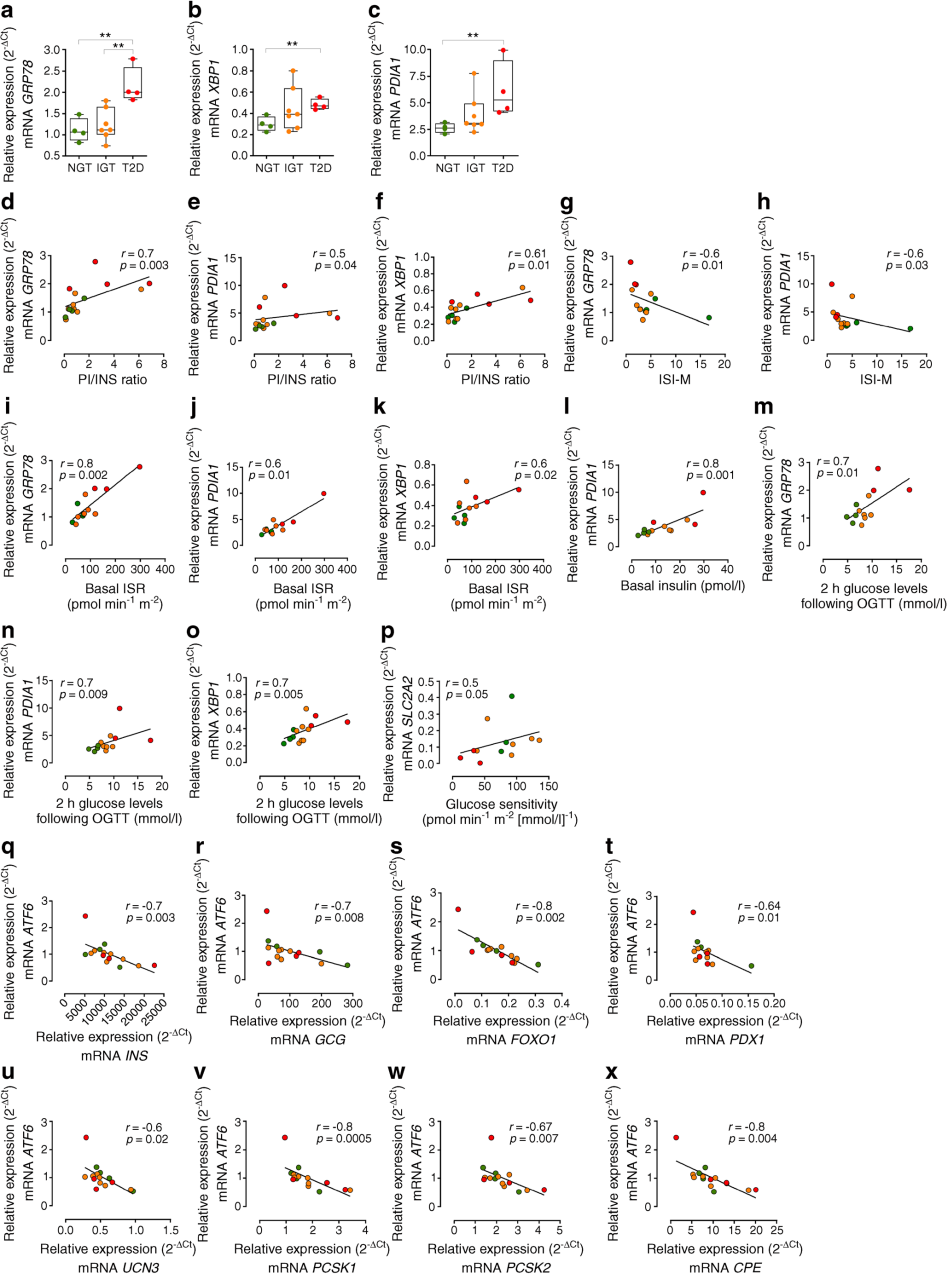
ER stress is associated with proinsulin/insulin expression and processing defects and with in vivo metabolic derangements. Real-time PCR expression analysis of *GRP78* (**a**), *XBP1* (**b**) and *PDIA1* (**c**) in pooled LCM pancreatic islets of *n*=4 NGT, *n*=7 IGT and *n*=4 type 2 diabetic participants. **p<0.01, one-way ANOVA and Tukey’s multiple comparisons post hoc test. (**d**–**p**) Graphs showing the correlation analysis in pancreatic islets of NGT, IGT and type 2 diabetic participants of *GRP78* (**d**), *PDIA1* (**e**) and *XBP1* (**f**) with PI/INS area ratio; *GRP78* (**g**) and *PDIA1* (**h**) with ISI-M; *GRP78* (**i**), *PDIA1* (**j**) and *XBP1* (**k**) with basal ISR (pmol min^−1^ m^−2^); *PDIA1* (**l**) with basal insulin (pmol/l); *GRP78* (**m**), *PDIA1* (**n**) and *XBP1* (**o**) with glucose levels 2 h after OGTT (mmol/l); and *SLC2A2* (**p**) with glucose sensitivity (pmol min^−1^ m^−2^ [mmol/l]^−1^). (**q**–**x**) Correlations of *ATF6* expression in pooled LCM pancreatic islets of NGT, IGT and type 2 diabetic participants with *INS* (**q**), *GCG* (**r**), *FOXO1* (**s**), *PDX1* (**t**), *UCN3* (**u**), *PCSK1* (**v**), *PCSK2* (**w**) and *CPE* (**x**). Green, NGT participants; orange, IGT participants; red, type 2 diabetic participants. Correlations included all participants with available metabolic/clinical measures and a valid RIN (≥5.0). For correlations, *p* and *r* values were obtained using Spearman correlation test. INS, insulin; PI, proinsulin; T2D, type 2 diabetic

In light of the association between metabolic alterations, increased beta cell proinsulin and proinsulin–insulin colocalisation in IGT and type 2 diabetic participants, we subsequently proceeded to investigate the putative molecular pathways underlying the observed defects. Intracellular expression alterations may depend on proinsulin misfolding, proinsulin processing alterations, enhanced ER stress and/or loss of beta cell phenotype. We analysed the expression of a set of selected genes (ESM Table 4) in pooled LCM islets (~60 islets/individual) obtained from frozen pancreatic tissue sections of NGT, IGT and type 2 diabetic participants (ESM Figs 5, 6) previously analysed for proinsulin and insulin in situ expression and distribution. We observed a significant upregulation of *GRP78* (also known as *HSPA5*) in type 2 diabetes compared with IGT and NGT pancreatic islets (*p*=0.004) (Fig. 3a) and a significant increase of x-box binding protein-1 (*XBP1*) and of *PDIA1* (also known as *P4HB*) in type 2 diabetes (*p*=0.02) (Fig. 3b,c), suggesting the activation of ER stress pathways. Since LCM pancreatic islet gene expression and proinsulin–insulin immunofluorescence analyses were performed on consecutive serial frozen sections, we correlated the expression of genes involved in ER stress, beta cell function and phenotype with in situ proinsulin–insulin staining measurements and participant clinical variables. The correlation matrix (ESM Fig. 7) revealed that *GRP78*, *PDIA1* and *XBP1* expression levels are positively correlated with increased proinsulin/insulin ratio (*GRP78*: *r*=0.7, *p*=0.003; *PDIA*: *r*=0.5, *p*=0.04; *XBP1*: *r*=0.61, *p*=0.01) (Fig. 3d–f), thus suggesting a putative link between ER stress and proinsulin and insulin expression and/or processing.

These results show that ER stress marker genes are significantly increased in type 2 diabetic and IGT pancreatic islets, and that this increase is closely correlated with altered proinsulin/insulin expression and alteration of insulin processing.

### In vivo insulin resistance and increased insulin demand are associated with ER stress

*GRP78*, *PDIA1* and *XBP1* in situ expression levels were significantly correlated with metabolic variables such as ISI-M (*GRP78*: *r*=−0.6, *p*=0.01; *PDIA1*: *r*=−0.6, *p*=0.03) (Fig. 3g,h), basal ISR (*GRP78*: *r*=0.8, *p*=0.002; *PDIA1*: *r*=0.6, *p*=0.01; *XBP1*: *r*=0.6, *p*=0.02) (Fig. 3i–k), basal insulin (*PDIA1*: *r*=0.8, *p*=0.001) (Fig. 3l) and 2 h glucose levels at OGTT (*GRP78*: *r*=0.7, *p*=0.01; *PDIA1*: *r*=0.7, *p*=0.009; *XBP1: r=0.7, p=0.005*) (Fig. 3m–o), denoting a close link between insulin resistance, consequent high basal insulin secretion and ER stress molecular markers. We also observed that expression levels of the ‘glucose sensor’ *SLC2A2*, which encodes GLUT2, are directly related to glucose sensitivity measured in vivo (*r*=0.5; *p*=0.05) (Fig. 3p).

The correlation matrix analysis also showed that the expression levels of *ATF6*, involved in ER stress mechanisms, were inversely associated with specific beta and alpha cell genes such as *INS* (*r*=−0.7, *p*=0.003), *GCG* (*p*=−0.7, *p*=0.008), *FOXO1* (*r*=−0.8, *p*=0.0002), *PDX1* (*r*=−0.64, *p*=0.01) and *UCN3* (*r*=−0.6, *p*=0.02) (Fig. 3q–u), and also with genes involved in proinsulin processing such as *PCSK1* (*r*=−0.8, *p*=0.0005), *PCSK2* (*r*=−0.67, *p*=0.007) and *CPE* (*r*=−0.8, *p*=0.004) (Fig. 3v–x), suggesting that activation of ER stress could be associated with the loss of beta cell identity and function.

Collectively, these results showed that ER stress marker genes are also significantly related to increased insulin demand and worsening of glucose metabolism in IGT and type 2 diabetes patients.

### Individual islet microdissection and phenotyping showed progressive alterations of proinsulin processing, ER stress and loss of beta cell identity during type 2 diabetes development

The gene expression analysis of LCM-pooled islets revealed a prominent alteration of ER stress-related genes and evidenced their correlation with in situ proinsulin–insulin immunofluorescence data and in vivo beta cell dysfunction occurring during progression to type 2 diabetes. However, by pooling and analysing multiple LCM pancreatic islets, the extraction of individual data is hampered by the high degree of heterogeneity among islets of the same pancreatic tissue section. To better investigate this heterogeneity and to study, at the islet level, the molecular mechanisms accompanying beta cell dysfunction during progression to type 2 diabetes, we studied the expression of 14 selected phenotypic and ER stress-related genes (see the ESM Methods section) together with individual in situ immunofluorescence insulin and proinsulin data (a total of 17 markers) of individual microdissected pancreatic islets obtained from NGT, IGT and type 2 diabetic individuals (ESM Fig. 8).

**Fig. 4:**
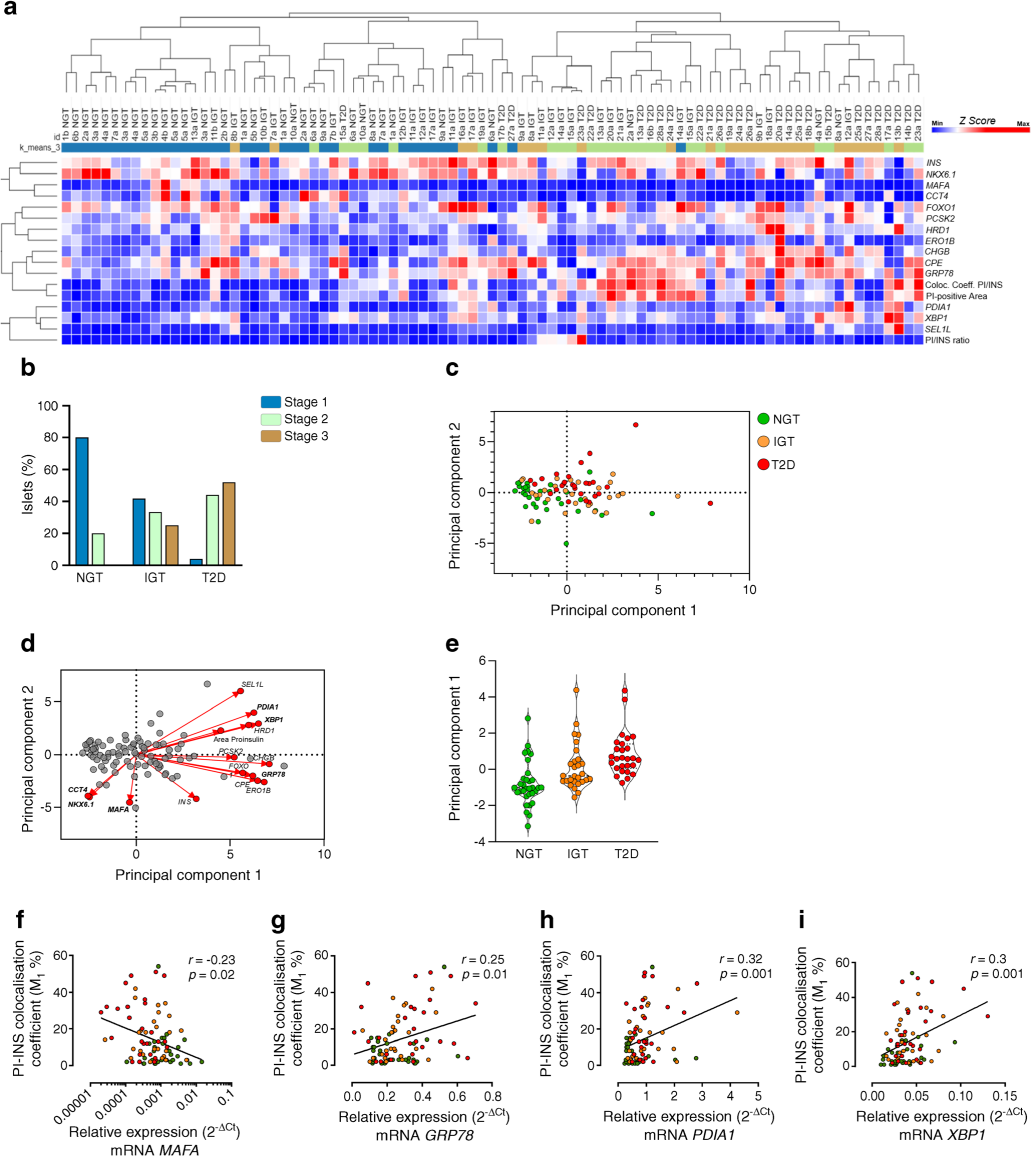
Analysis of individual human pancreatic islets reveals a direct association between proinsulin processing, ER stress and beta cell dysfunction during in vivo metabolic alterations. (**a**) Hierarchical clustering analysis of genes associated with ER stress, beta cell function and proinsulin/insulin in situ staining variables of individual pancreatic islets of *n*=3 NGT, *n*=3 IGT and *n*=3 type 2 diabetic participants. Colour key indicates *z* score values from the least expressed (dark blue) to the most expressed (dark red). (**b**) Histogram graph showing individual islet staging classification based on *k*-means clustering (*n*=3) values, which discriminates the individual islet profiles into three different progression stages (Stage 1 [normal], Stage 2 [intermediate dysfunction], Stage 3 [full dysfunction]; blue, green or brown colour coded, respectively). (**c**) PCA plot shows three clusters of analysed samples in which the individual islets are represented by green (NGT), orange (IGT) and red (type 2 diabetes) and projected into a 2D space (components 1 and 2). (**d**) PCA biplot showing principal component scores and loading values, which determine most of the directionality of individual islet progression based on the set of evaluated markers. (**e**) Graph showing principal component 1 values which determine the individual islet progression exclusively based on the following markers: *MAFA*, *NKX6.1*, *CCT4*, *PDIA1*, *XBP1*, *GRP78*. (**f**–**i**) Correlations of beta cell identity gene *MAFA* (**f**) (plotted on log_10_ axis) or ER-related genes *GRP78* (**g**), *PDIA1* (**h**) and *XBP1* (**i**) with immunofluorescence in situ PI/INS variables. NGT participants, green; IGT participants, orange; and type 2 diabetic participants, red; *p* and *r* values were obtained using Spearman *r* test. INS, insulin; PI, proinsulin; T2D, type 2 diabetic

As observed in previous immunofluorescence in situ analyses (Fig. 2), we confirmed that the proinsulin–insulin colocalisation coefficient, proinsulin-positive area and proinsulin/insulin area ratio were increased in pancreatic islets of IGT and type 2 diabetic individuals compared with NGT individuals (*p*<0.01) (ESM Fig. 9a–c). Gene expression analysis on individual islets using a hierarchical clustering approach showed that the 88 pancreatic islets were clearly subdivided into two main clusters of individual islet profiles, separating NGT from IGT and type 2 diabetes (Fig. 4a). We observed a significant overlap between IGT and type 2 diabetic islets that were included in the same main cluster; however, a downstream node separated IGT and type 2 diabetic islets into two sub-clusters, as expected. Overall, we observed a progressive increase of altered insulin processing variables together with ER stress markers, and a decrease of beta cell phenotypic markers (Fig. 4a). The *k*-means clustering allowed us to separate individual islet profiles into three different stages, approximating the progression from functional to dysfunctional profiles over time (Stage 1 [normal], Stage 2 [intermediate dysfunction], Stage 3 [full dysfunction]), thus supposedly characterising individual pancreatic islets during disease progression. Most of the NGT islets were classified as Stage 1 (80.0%), significantly fewer as Stage 2 (20.0%) and none as Stage 3 (0%). In IGT, Stage 1 islets decreased (41.7%) while Stage 2 (33.0%) and Stage 3 (25%) islets increased. Conversely, type 2 diabetic islets were mostly classified as Stage 3 (52.0%) and Stage 2 (44.0%), while only 4% of islets were classified as Stage 1 (Fig. 4b). Principal component analysis (PCA) confirmed the different characteristics of NGT islets compared with IGT and type 2 diabetes and further showed the progression of islet demise based on our set of markers (Fig. 4c). Of interest, the major features determining islet progression from NGT to IGT and type 2 diabetes were ER stress genes, such as *PDIA1*, *XBP1* and *GRP78*, while *MAFA*, *NKX6.1* and *CCT4* represented the main determinants of islet functional phenotype by directing the distribution of individual islets in the opposite direction (Fig. 4d). A PCA plot bar graph (Fig. 4e) based on the expression levels of *MAFA*, *NKX6.1*, *CCT4*, *XBP1* and *GRP78*, as well as *PDIA1* (ESM Fig. 10), shows a clearer division between the three groups, highlighting the progression from NGT to IGT and then type 2 diabetes.

Collectively, we observed a progressive decrease in pivotal beta cell/islet phenotype functional markers, along with a progressive increase in ER stress-related components; these defects were accompanied by an increase in proinsulin-positive area, proinsulin/insulin ratio and proinsulin–insulin colocalisation, thus confirming the close relationship between proinsulin/insulin processing, ER stress and functional phenotype-related genes.

As a final confirmation, we performed a correlation matrix analysis of molecular and immunohistochemical individual islet features (ESM Fig. 11). We observed a negative correlation between the beta cell phenotype-related gene *MAFA* and proinsulin–insulin colocalisation rate (Fig. 4f), while positive correlations were detected between ER stress-related genes and proinsulin–insulin colocalisation rate (Fig. 4g–i). Additional significant correlations between beta cell phenotype-related genes, ER markers and in situ proinsulin/insulin data corroborated the link between ER stress, phenotype and proinsulin/insulin defects (ESM Fig. 12).

Collectively, our data showed that metabolic stress occurring during type 2 diabetes progression induces significant changes in ER stress, leading to impaired insulin synthesis in situ, loss of beta cell identity and beta cell dysfunction.

## Discussion

Our study demonstrates that in the natural history of type 2 diabetes, progressive beta cell dysfunction is characterised by a progressive increase in ER stress accompanied by an altered insulin processing, finally leading to a loss of beta cell phenotype.

This is the first study in which ER stress markers, insulin synthesis and beta cell identity have been linked to in vivo specific metabolic features of insulin secretion and sensitivity in the same individual in a cohort of participants with different metabolic conditions (NGT, IGT and overt type 2 diabetes), allowing us to obtain progressive snapshots of the natural history of type 2 diabetes.

Specifically, we found that the transition from non-diabetic insulin-sensitive to insulin-resistant is characterised by an increased insulin demand initially leading to in situ changes in the expression of ER stress/UPR-related genes and a consequent alteration in insulin processing. Indeed, the increase in the proinsulin area suggests an increase in proinsulin production to overcome high insulin demand and/or a defect in insulin processing, which may cause its accumulation and secondary mislocalisation. Our findings suggest that the alteration of insulin processing in situ starts in the IGT state and that this molecular feature reflects in vivo impairment of beta cell function.

It is worth noting that beta cell glucose sensitivity measured in vivo is also directly linked to the expression of *SLC2A2* (encoding GLUT2) in LCM-captured pancreatic islets, suggesting that higher levels of *SLC2A2* are correlated with a better beta cell function. This result endorses the role of in vivo-measured beta cell glucose sensitivity during OGTT or MMT as a valuable and sensitive estimation of beta cell molecular function [17, 18].

In particular, in the progression towards type 2 diabetes, impaired beta cell function is linked to higher insulin demand driven by insulin resistance. Previous studies have shown that this increased beta cell workload is characterised by protein misfolding and accumulation, leading to ER stress. Indeed, it has been demonstrated that elevated proinsulin circulating levels [19–22] associated with its increased biosynthesis represent the primary driver of ER protein load in beta cells, causing accumulation of misfolded proinsulin [23–25] which leads to adaptive UPR and, if unresolved, to terminal UPR and ER stress [25–28]. Our findings highlight the crucial role of insulin resistance in the impairment of proinsulin synthesis and/or processing, confirming this evidence in a model of increased beta cell workload (induced by acute surgical removal of 50% of beta cell mass against a background of insulin resistance) [5].

Further, we demonstrate that proinsulin/insulin defects and ER stress markers progressively increase in the transition from NGT to IGT and that these changes are directly linked to the initial loss of beta cell identity. In particular, we observed that the expression of *NKX6.1* and *MAFA*, previously associated with beta cell dedifferentiation [29–31], was reduced in pancreatic islets from IGT compared with control individuals, while *FOXO1*, a key transcription factor involved in beta cell function and identity [29–31], was upregulated (ESM Fig. 10h), suggesting a final attempt to compensate for high metabolic stress to preserve beta cell function.

In addition, when we analysed the transition from IGT to diabetes, we observed an additional reduction in the expression of *NKX6.1* and *MAFA*. This confirms previous in vitro findings in diabetic individuals with a downregulation of *FOXO1*, which suggests the impaired capacity of type 2 diabetic islets to compensate for metabolic requirements and a final propensity to acquire a dedifferentiated phenotype [29–32]*.*

More importantly, we found that chronic increase in beta cell workload (increased insulin demand and insulin resistance) induces ER stress and impairment of the beta cell secretory machinery, as shown in the altered expression of in situ proinsulin/insulin ratio.

Overall, these data also confirm and explain previous in vitro observations showing that long-term overexpression of misfolding-prone mutant proinsulin or increased beta cell stress stimuli induced insulin secretion defects, increased ER stress markers and led to loss of beta cell identity [33, 34].

Of all the mechanisms involved in beta cell dysfunction [35], ER stress represents a determinant link between altered insulin processing and loss of beta cell identity and dysfunction in type 2 diabetes. Previous studies have shown increased ER volume density and beta cell apoptosis in type 2 diabetes, with increased beta cell susceptibility to high glucose-induced ER stress in islets isolated from type 2 diabetic individuals, suggesting that ER stress is one of the primary processes involved in beta cell dysfunction [9].

Our study design presents several advantages. First, we studied, in the same participants, both in situ and in vivo functional defects in the beta cell secretory machinery, including beta cell glucose sensitivity (a measure of the amount of secreted insulin for any specific glucose concentration) [17, 36, 37]. All individuals were evaluated not only through anamnesis and HbA_1c_, but also using the gold standard OGTT and 4 h MMT, thus allowing us to determine insulin secretion, insulin resistance and measures of beta cell function in vivo (basal insulin secretion, beta cell glucose sensitivity). Finally, we took advantage of the elevated inter-islet heterogeneity to observe the changes in phenotypes and characteristics that take place in the course of worsening glucose tolerance in individual islets.

Given the close correlation between clinical and in situ markers for each individual participant and the high heterogeneity among islets in the same glucose tolerance group, we used an unprecedented individual islet phenotyping approach. This approach collects morphological/immunohistochemical and gene expression datasets to evaluate multiple aspects of individual islets and to detect changes in beta cell identity occurring during the late stage of beta cell failure. Using individual islet LCM analysis, we were able to detect significant changes not previously observed using pooled islet analysis. This can be explained by the great heterogeneity between different islets of the same tissue section. The latter can be an important confounding factor and lead to non-significant results in pooled islet analysis if the measured signal is not strong enough to overcome the source of variance arising from the naturally occurring heterogeneity of the islets. In fact, pancreatic islet heterogeneity is a well-recognised feature of endocrine pancreas physiology [38] and should be taken into account also in the context of diabetes [39, 40].

Using this individual islet analysis approach, we were able to stratify pancreatic islets into three different stages from functional to dysfunctional. This allowed us to show that in the natural history of diabetes there is a progressive transition from mature and competent islets to islets with a dysfunctional profile and that these phenomena are directly linked to impairment of beta cell function in vivo.

We observed that in the transition from impaired glucose tolerance to diabetes, the percentage of individual islets showing increased ER stress markers, altered proinsulin processing and decreased beta cell phenotype markers increases significantly. These individual islets, which are highly represented in IGT and type 2 diabetic individuals, have a similar phenotype and molecular profile that is completely different from that of the individual NGT islets, suggesting progressive damage. Further, we were able to reconstruct, at the islet level, the progressive damage observed in islets from different individuals, classified on the basis of their glucose tolerance, suggesting that the alterations described in pooled islets from different individuals occur in each single individual progressively, and only when the islets with dysfunctional profiles become predominant does the clinical worsening of glucose metabolism become evident.

Overall, we found that in the progression from NGT to IGT to type 2 diabetes the individual islets displayed: (1) a progressive decrease in markers associated with beta cell identity, together with chaperones and oxidoreductase factors; (2) a progressive increase in ER stress marker genes; (3) an increase in insulin processing machinery components in IGT islets as an attempt to compensate for the increase in insulin demand; (4) a progressive increase of altered in situ proinsulin expression and intracellular localisation.

In conclusion, the progression towards type 2 diabetes is characterised by changes in the expression of ER stress/UPR-related genes, and consequent defects in intra-islet insulin synthesis leading to loss of beta cell identity and dysfunction. This suggests that these pre-existing defects in the beta cell secretory machinery may be pivotal in leading to beta cell dedifferentiation and insulin deficiency in type 2 diabetes. A better understanding of these mechanisms in humans will allow us to understand which functional step fails in response to insulin resistance and consequent increased insulin demand, and at which stage this progression towards beta cell dedifferentiation is reversible. Thus, therapeutic strategies aiming at reducing beta cell workload may prevent ER stress and in the long run beta cell dedifferentiation, and consequently delay beta cell failure in type 2 diabetes.

## Supplementary information

ESM (PDF 1408 kb)

## Data Availability

All data are included in the present study or are available upon request.

## Abbreviations

ER: Endoplasmic reticulum
IGT: Impaired glucose tolerant
ISI-M: Matsuda insulin sensitivity index
ISR: Insulin secretion rate
LCM: Laser capture microdissection
MMT: Mixed meal test
NGT: Normal glucose tolerant
PCA: Principal component analysis
RIN: RNA integrity
UPR: Unfolded protein response
