# Supplementary material for: Intra-islet insulin synthesis defects are associated with endoplasmic reticulum stress and loss of beta cell identity in human diabetes
Source: Diabetologia. 2022 Oct 25;66(2):354–66. doi: 10.1007/s00125-022-05814-2 (PMC9807540; doi:10.1007/s00125-022-05814-2)
Supplement: Supplementary file 1 — (PDF 1408 kb) [file 125_2022_5814_MOESM1_ESM.pdf]

ELECTRONIC SUPPLEMENTARY MATERIAL (ESM)

ESM METHODS

Subjects and metabolic screening

Eighteen patients [12 females; 6 males; mean age 67.7±12.2 (yrs. ± S.D.)] undergoing pylorus-preserving pancreatoduodenectomy were recruited from January 2017 to July 2019 at the Digestive Surgery Unit and studied at the Centre for Endocrine and Metabolic Diseases unit (both at the Agostino Gemelli University Hospital, Rome, Italy. All underwent a complete metabolic screening including oral glucose tolerance test (OGTT) and mixed meal test (MMT) (ESM Methods). The study protocol (ClinicalTrials.gov Identifier: NCT02175459) was approved by the local ethics committee (P/656/CE2010 and 22573/14) (Rome, Italy) and all participants provided written informed consent, which was followed by a comprehensive medical evaluation.

Oral Glucose Tolerance Test. A standard 75 g oral glucose tolerance test was performed with measurement of glucose, insulin and C-peptide at 0, 30, 60, 90, 120 min after glucose load. Based on the pre-surgery OGTT results, we classified the patients according to the ADA classification (American Diabetes Association, 2019): subjects whose 2 h post glucose load was below 7.8 mmol/l were defined as normal glucose tolerant (NGT, n=5), subjects whose 2 h post glucose load was 7.8–10.9 mmol/l were defined as impaired glucose tolerant (IGT, n=9) and subjects whose 2 h post glucose load was higher than 11.1 mmol/l and with known history of type 2 diabetes of over two years and/or on anti-diabetic medications, were defined as diabetic (T2DM, n=4).

Mixed meal test. A mixed meal test was performed, as previously described [1, 2]. Patients were instructed to consume a meal of 830 kcal (107 kcal from protein, 353 kcal from fat, and 360 kcal from carbohydrates) within 15 min. Blood samples were drawn twice in the fasting state and at 30 min intervals over the following 240 min (sample time 0’, 30’, 60’, 90’, 120’, 150’, 180’, 210’ and 240’) for the measurement of plasma glucose, insulin, C-peptide. Insulin levels were determined using a commercial RIA kit (Medical System, Immulite DPC, Los Angeles, CA). Plasma glucose concentrations were determined by the glucose oxidase technique, using a glucose analyser (Beckman Instruments, Palo Alto, CA, USA). Plasma C-peptide was measured by auto DELPHIA automatic fluoroimmunoassay (Wallac, Turku, Finland), with a detection limit of 17 pmol/L.

Calculations. During the OGTT and MMT, insulin secretion was derived from C-peptide levels by deconvolution. Beta-cell Glucose Sensitivity (βCGS), i.e., the slope of the relationship between insulin secretion and glucose concentration, was estimated from the OGTT or MMT by modelling, as previously described[3–5]. Matsuda index [6] was calculated as index of whole-body insulin sensitivity based on insulin and glucose values obtained from the OGTT.

Surgical procedures

Indications for surgery were: periampullary tumours, pancreatic intraductal papillary tumours, mucinous cystic neoplasm of the pancreas, non-functional pancreatic neuroendocrine tumours. Only patients with normal cardiopulmonary and kidney function, as determined by medical history, physical examination, electrocardiography, estimated glomerular filtration rate, and urinalysis were included. Altered serum lipase and amylase levels prior to surgery, as well as morphologic criteria for pancreatitis, were considered exclusion criteria. Patients with severe obesity (BMI> 40), uncontrolled hypertension and/or diabetes (with HbA1c ≥ 58 mmol/mol (7.5%) and/or hypercholesterolemia were also excluded.

Pancreatoduodenectomy was performed according to the pylorus preserving technique. Briefly, the pancreatic head, the entire duodenum, common bile duct, and gallbladder were removed en-bloc, leaving a functioning pylorus intact at the gastric outlet. All adjacent lymph nodes were carefully removed. The continuity of the gastrointestinal tract was restored by an end-to-side pancreatojejunostomy. Further downstream, an end-to-side hepaticojejunostomy and an end-to-side pylorojejunostomy were performed. The volume of pancreas removed during the surgery is constant (~50%), as previously reported by Schrader et al. [2]. A pancreatic sample was collected during the surgery, from the downstream edge of the surgical cut. Pancreas samples were snap frozen in liquid nitrogen and then stored at -80 until analysis.

Proinsulin-Insulin immunofluorescence of human pancreatic sections and imaging analysis

Frozen pancreatic 7 µm thick tissue sections were used. After one hour of drying at room temperature (RT), the sections were fixed in paraformaldehyde (PFA) 4% in PBS for 20 minutes at RT. Sections were then washed three times in 0,1% PBS-tween w/ Ca^{2+} Mg^{2+} for 5 min at RT. To unmask the antigens, pancreatic sections were subjected to antigen retrieval using 10 mM citrate buffer pH 9.0 at 70°C for 40 minutes. Sections were washed three times in 0,1% PBS-tween w/ Ca^{2+} Mg^{2+} for 5 min at RT. Subsequently, sections were incubated with PBS 1X supplemented with 1% Bovine Serum Albumin (BSA, cat. A1470-25G, Sigma

Aldrich, St. Louis, MO, USA) to reduce non-specific reactions. Then, sections were incubated with primary antibodies, polyclonal Guinea Pig anti-human Insulin (cat. A0564 - Agilent Technologies, Santa Clara, CA, USA) diluted 1:2000 and Mouse monoclonal anti-human Proinsulin (cat. GS9A8–Developmental Study Hybridoma Bank, Iowa City, IA-USA (epitope: B-C junction of proinsulin spanning aa 26-37)[7–9] diluted 1:100 in PBS 1X supplemented with 1% BSA overnight in damp chamber at 4°C. The next day, sections were washed three times in 0,1% PBS-tween w/ Ca^{2+} Mg^{2+} 5 min each at RT and subsequently incubated with secondary antibodies, goat anti-guinea pig Alexa-Fluor 488 conjugate (cat. A11073, Molecular Probe, Thermo Fisher Scientific, Waltham, MA, USA) diluted 1:500 in PBS 1X and goat anti-mouse Alexa-Fluor 594 conjugate (cat. A11032, Molecular Probe, Thermo Fisher Scientific, Waltham, MA, USA) diluted 1:500 in PBS 1X for 1h at RT. After the washes sections were counterstained with 4′,6-Diamidino-2-phenylindole dihydrochloride (DAPI, cat. D8517, Sigma-Aldrich) diluted 1:3000 in PBS 1X for 5 minutes, and then mounted with Vectashield antifade medium (cat. H-1000, Vector Laboratories, Burlingame, CA, USA) and analysed immediately or stored at +4°C until ready for confocal microscope fluorescence image analysis.

Images were acquired as a single stack focal plane or in z-stack mode capturing multiple focal plane images. Sections were scanned and images acquired at 40x magnification. The same confocal microscope setting parameters (laser power, photomultiplier voltage gain and offset values, pinhole value) were applied to all stained sections before image acquisition to uniformly collect detected signal related to each fluorescence channel. Image analysis was performed using Volocity 6.3 software (Perkin Elmer, Waltham, MA, USA) on each individual islet to measure colocalization coefficient (M1), islet area (μm^2), PI and INS positive area. The islet area (μm^2) was calculated taking into consideration the value (sum area) which represents the total number of objects that identify the total area of islet on the basis of the region of interest (ROI). A background threshold filter was uniformly applied to all processed images before the evaluation of specific parameters. After setting manual thresholds based on the positivity of the signals to be analyzed, the PI and INS positive area was calculated. The PI/INS ratio was calculated by dividing the PI signal positive area and the INS positive area, normalized by the total area of islet.

The percentage of colocalization coefficient between PI and INS was calculated through the Colocalization Coefficient M1 (Mander's coefficient) that considers the percentage of pixels (or voxels in case of volume) of a given channel, in our case the INS, which overlaps to total pixels (or voxels) related to the other channel, in our case the PI. Of note, Mander's coefficient is independent of absolute signal as it measures the fraction of

one protein that colocalizes with a second protein where M represents the fraction (reported in percentage) of colocalizing pixels channel-1/channel-2 on total channel-1 pixels.

Pixel intensity distribution analysis was performed using Image J (v1.53c)(imagej.nih.gov) plot profile function on single fluorescence channel exported images.

Laser capture microdissection (LCM) of human pancreatic islets

Pancreatic human tissue samples from n=5 NGT, n=9 IGT and n=4 T2D living donors (**Table 1**) were embedded in Tissue-Tek O.C.T. compound (Sakura Finetek Europe, The Netherlands) and immediately frozen in liquid nitrogen, then stored at -80°C. Then, 7 µm thick tissue sections were cut from frozen O.C.T. blocks using a cryostat (Leica CM1950UV). Sections were fixed in 70% ethanol for 30 s, dehydrated in 100% ethanol for 1 min, in 100% ethanol for 1 min, in xylene for 5 min and finally air-dried for 5 min. Laser capture microdissection (LCM) was performed using an Arcturus XT Laser-Capture Microdissection system (Arcturus Engineering, Mountain View, CA, USA) by melting thermoplastic films mounted on transparent LCM caps (cat. LCM0214 - ThermoFisher Scientific, Waltham, MA, USA) on specific islet areas. Human pancreatic islets were subsequently visualized through islet autofluorescence for LCM procedure. Thermoplastic films containing microdissected cells were incubated with 10 µl of extraction buffer (cat. kit0204 - ThermoFisher Scientific, Waltham, MA, USA) for 30 min at 42 ◦C and kept at −80°C until RNA extraction. Each microdissection was performed within 30 min from the staining procedure. Overall ~60 microdissected pancreatic islets from each case were analysed for the global molecular analysis.

Gene expression analysis of pooled and individual LCM pancreatic islets

Total RNA was extracted from each LCM sample using PicoPure RNA isolation kit Arcturus (cat. kit0204 - ThermoFisher Scientific, Waltham, MA, USA) following manufacturer's procedure. Briefly, cellular extracts were mixed with 12.5 µl of Ethanol (100%) and transferred to the purification column filter membrane. DNase treatment was performed using RNase-Free DNase Set (cat. 79254 - Qiagen, Hilden, Germany). Total RNA was eluted in 11 µl of DNase/RNase-Free Water and LCM captures deriving from human sample were pooled and subjected to a subsequent concentration through Savant SpeedVac SC100 centrifugal evaporator. Agilent 2100 Bioanalyzer technology with RNA Pico chips (cat. 5067-1513 Agilent Technologies, Santa Clara, CA, USA) was performed for each RNA sample, to evaluate RNA Integrity Number (RIN) and concentration, by excluding samples with RIN<5.0.

To analyse gene expression associated with ER stress, folding, processing of proinsulin and beta-cell function and dysfunction, a reverse transcriptase reaction was performed on each RNA sample extracted (500 pg) from microdissected islet endocrine cells from each donor using SuperScript™ VILO™ cDNA Synthesis Kit (cat. 11754050- ThermoFisher Scientific, Waltham, MA, USA). cDNA deriving was then amplified using TaqMan PreAmp Master Mix (cat. 4488593, ThermoFisher Scientific, Waltham, MA, USA) following manufacturer's instructions. Real-Time PCR analysis was performed using TaqMan gene expression assays using the primers (see key resource table) and SensiFast Probe Lo-ROX Kit (cat.# BIO-84020, Bioline) following manufacturer's recommendation. Data were collected and analysed through Expression Suite software 1.0.1 (ThermoFisher Scientific, Waltham, MA, USA) using $2^{-\Delta Ct}$ or $2^{-\Delta\Delta Ct}$ method. Data analysis was performed using ViiA7™ RUO software to collect data and Expression Suite 2.1 software (both Thermo Fisher Scientific) to evaluate amplification plot efficiency and to export Ct values. Analysis was performed using $2^{-\Delta CT}$ following normalization with *ACTB* and *GAPDH*.

The phenotypic characterization of individual pancreatic islets was performed using two consecutive frozen pancreatic sections for each subject (**ESM Fig. 8**). In Section#1 we performed a PI-INS staining to characterize the morphological profile of each individual islet in order to evaluate the in-situ levels of PI and INS. After carrying out the image analysis as described above, we mapped the localisation and distribution of individual pancreatic islets by capturing low magnification images of the entire section. In Section#2 we isolated the previously mapped individual islets to analyse genes expression and to investigate putative molecular alterations associated with PI-INS expression and processing alterations. The laser capture microdissection procedure was performed as described above, isolating an individual islet at a time (**ESM Fig. 8**). Overall, we selected and evaluated 17 markers including 14 genes related to beta-cell phenotype and ER stress (*INS, NKX6.1, MAFA, CCT4, FOXO1, PCSK2, HRD1, ERO1B, CHGB, CPE, GRP78, PDIA1, XBP1, SEL1L*), and 3 individual in situ immunofluorescence PI and INS data (PI-INS Coloc. Coeff. M1, PI area and PI/INS area ratio) each individual microdissected pancreatic islet obtained from n=3 NGT, n=3 IGT and n=3 T2D individuals.

The whole eluate of RNA extracted from each individual microdissected islet (approximately 8.8 µl) using PicoPure RNA isolation kit (cat. kit0204 - ThermoFisher Scientific, Waltham, MA, USA), was subjected to a reverse transcriptase reaction using SuperScript™ VILO™ cDNA Synthesis Kit (cat. 11754050- ThermoFisher Scientific, Waltham, MA, USA) to analyse genes expression in individual pancreatic islets. The total volume of cDNA was then preamplified using TaqMan PreAmp Master Mix (cat. 4488593, ThermoFisher

Scientific, Waltham, MA, USA) by preparing the preamplification pool using selected TaqMan Gene expression assays by diluting each 20X TaqMan gene expression assays to obtain a final concentration of each assay equals to 0.5X. The preamplification product was diluted 1:5 in 0.1X Tris-EDTA pH 8.0. Real-Time PCR analysis was performed using TaqMan gene expression assays and SensiFast Probe Lo-ROX Kit (cat.# BIO-84020, Bioline) following manufacturer's recommendation.

References

1. Muscogiuri G, Sorice GP, Mezza T, et al. (2013) High-normal TSH values in obesity: is it insulin resistance or adipose tissue's guilt? Obesity (Silver Spring) 21:101–106. doi: 10.1002/oby.20240

2. Schrader H, Menge BA, Breuer TGK, et al. (2009) Impaired glucose-induced glucagon suppression after partial pancreatectomy. J Clin Endocrinol Metab 94:2857–2863. doi: 10.1210/jc.2009-0826

3. Mari A, Schmitz O, Gastaldelli A, et al. (2002) Meal and oral glucose tests for assessment of beta -cell function: modeling analysis in normal subjects. Am J Physiol Endocrinol Metab 283:E1159–66. doi: 10.1152/ajpendo.00093.2002

4. Mari A, Tura A, Natali A, et al. (2010) Impaired beta cell glucose sensitivity rather than inadequate' ' compensation for insulin resistance is the dominant defect in glucose intolerance. Diabetologia 53:749–756. doi: 10.1007/s00125-009-1647-6

5. Mezza T, Sorice GP, Conte C, et al. (2016) β-Cell Glucose Sensitivity Is Linked to Insulin/Glucagon Bihormonal Cells in Nondiabetic Humans. J Clin Endocrinol Metab 101:470–475. doi: 10.1210/jc.2015-2802

6. Matsuda M, DeFronzo RA (1999) Insulin sensitivity indices obtained from oral glucose tolerance testing: comparison with the euglycemic insulin clamp. Diabetes Care 22:1462–1470. doi: 10.2337/diacare.22.9.1462

7. Asadi A, Bruin JE, Kieffer TJ (2015) Characterization of antibodies to products of proinsulin processing using immunofluorescence staining of pancreas in multiple species. J Histochem Cytochem 63:646–662. doi: 10.1369/0022155415576541

8. Madsen OD (1987) Proinsulin-specific monoclonal antibodies. Immunocytochemical application as beta-cell markers and as probes for conversion. Diabetes 36:1203–1211. doi: 10.2337/diab.36.10.1203

9. Rodriguez-Calvo T, Zapardiel-Gonzalo J, Amirian N, et al. (2017) Increase in Pancreatic Proinsulin and Preservation of β-Cell Mass in Autoantibody-Positive Donors Prior to Type 1 Diabetes Onset. Diabetes 66:1334–1345. doi: 10.2337/db16-1343

ESM TABLES

ESM Table 1. Reagents and Resources Table

**REAGENT or RESOURCE**	**SOURCE**	**IDENTIFIER**
Antibodies and reagent for IHC		
Polyclonal Guinea Pig Anti-Insulin	Agilent/Dako	A0564, RRID:AB_2617169
Monoclonal Mouse Anti-Proinsulin	DSHB	GS9A8, RRID:AB_532383
Goat Anti-Mouse IgG (H+L) Highly Cross-adsorbed Antibody, Alexa Fluor 594 Conjugated	Thermo Fisher Scientific	A-11032, RRID:AB_2534091
Goat anti-Guinea Pig IgG (H+L) Highly Cross-Adsorbed Antibody, Alexa Fluor 488	Thermo Fisher Scientific	A-11073, RRID:AB_2534117
Paraformaldehyde 4%	Sigma Aldrich	
DAKO PAP pen	Agilent/Dako	S2002
PBS w/o Ca2+ Mg2+	Gibco	14040-091
Bovine Serum Albumin	Sigma Aldrich	A1470-25G
Vectashield	Vector Laboratories	H-1000
Dako Fluorescence Mounting Medium	Agilent/Dako	S3023
DAPI	Sigma Aldrich	D8517
Citrate buffer:		
0.1 M Citric Acid Monohydrate	Sigma Aldrich	C1909
0.1M Sodium Citrate Tribasic Dihydrate	Sigma Aldrich	S4641
Chemicals		
Absolute Ethanol	VWR	1.009.862.500
Xilene	Merck	28975325
Hematoxylin	Sigma Aldrich	MHS31
PicoPure RNA isolation kit Arcturus	Thermo Fisher Scientific	kit0204
RNase-Free DNase Set	Qiagen	79254
Agilent 2100 Bioanalyzer technology with RNA Pico chips	Agilent Technologies	5067-1513
SuperScript™ VILO™ cDNA Synthesis Kit	Thermo Fisher Scientific	11754050-
SensiFast Probe Lo-ROX Kit	Aurogene s.r.l	BIO-84020
LCM caps	Thermo Fisher Scientific	LCM0214
Direct-zol RNA Miniprep Kit	Zymo Research	R202
Taq Man Preamp Master mix	Thermo Fisher Scientific	4488593
TaqMan assays		
Endogenous control human GAPDH	Thermo Fisher Scientific	4333764
Endogenous control human ACTB	Thermo Fisher Scientific	4333762
Endogenous control human B2M	Thermo Fisher Scientific	4333766
Taq Man gene expression assay INS	Thermo Fisher Scientific	Hs02741908_m1
Taq Man gene expression assay GCG	Thermo Fisher Scientific	Hs00174967_m1
Taq Man gene expression assay STT	Thermo Fisher Scientific	Hs00174949_m1
Taq Man gene expression assay PDX1	Thermo Fisher Scientific	Hs00236830_m1
Taq Man gene expression assay FOXO1	Thermo Fisher Scientific	Hs01054576_m1

Taq Man gene expression assay MAFA	Thermo Fisher Scientific	Hs01651425_s1
Taq Man gene expression assay NKX6.1	Thermo Fisher Scientific	Hs00232355_m1
Taq Man gene expression assay SEL1L	Thermo Fisher Scientific	Hs01071406_m1
Taq Man gene expression assay AMY2A	Thermo Fisher Scientific	Hs00420710_g1
Taq Man gene expression assay HRD1/SYVN1	Thermo Fisher Scientific	Hs00381211_m1
Taq Man gene expression assay ABCC8	Thermo Fisher Scientific	Hs01093752_m1
Taq Man gene expression assay ERP27	Thermo Fisher Scientific	Hs00929901_m1
Taq Man gene expression assay UCN3	Thermo Fisher Scientific	Hs07289310_m1
Taq Man gene expression assay SLC2A1	Thermo Fisher Scientific	Hs01096908_m1
Taq Man gene expression assay ACLY	Thermo Fisher Scientific	Hs00982738_m1
Taq Man gene expression assay IAPP	Thermo Fisher Scientific	Hs00169095_m1
Taq Man gene expression assay PC1/PCSK1	Thermo Fisher Scientific	Hs01026107_m1
Taq Man gene expression assay PC1/PCSK1	Thermo Fisher Scientific	Hs00159922_m1
Taq Man gene expression assay CPE	Thermo Fisher Scientific	Hs00175676_m1
Taq Man gene expression assay ERO1B	Thermo Fisher Scientific	Hs00219877_m1
Taq Man gene expression assay DDIT3	Thermo Fisher Scientific	Hs01090850_m1
Taq Man gene expression assay HSPA5	Thermo Fisher Scientific	Hs99999174_m1
Taq Man gene expression assay XBP1	Thermo Fisher Scientific	Hs00231936_m1
Taq Man gene expression assay EIF2AK3	Thermo Fisher Scientific	Hs00984003_m1
Taq Man gene expression assay ERN1	Thermo Fisher Scientific	Hs00980095_m1
Taq Man gene expression assay ATF6	Thermo Fisher Scientific	Hs00232586_m1
Taq Man gene expression assay CHGB	Thermo Fisher Scientific	Hs01084631_m1
Taq Man gene expression assay LMNB1	Thermo Fisher Scientific	Hs01059210_m1
Taq Man gene expression assay P4HB	Thermo Fisher Scientific	Hs01050257_m1
Taq Man gene expression assay TROVE2	Thermo Fisher Scientific	Hs01567394_m1
Taq Man gene expression assay CCT4	Thermo Fisher Scientific	Hs00272345_m1
Taq Man gene expression assay S100A9	Thermo Fisher Scientific	Hs00610058_m1
Software		
Expression Suite software 1.0.1	Thermo Fisher Scientific	
Leica TCS SP5 confocal laser scanning microscope system	Leica Microsystems	
Laser Advanced fluorescence	Leica Microsystems	
Volocity 3D Image analysis Software	Perkin Elmer	
Graph Pad Prism 6 and 8	Prism	

ESM Table 2. Table showing RNA integrity Number (RIN, scale 0-10) of total RNA extracted from pooled LCM islets captured from frozen pancreatic tissue sections of NGT, IGT and T2D subjects. In addition, type of analysis performed on each individual case is also reported (i.e. histology, pooled islet expression and/or individual islet expression analysis). Exclusively the samples with RIN ≥ 5.0 (highlighted in green) were considered for genes expression analysis of pooled islets, resulting in n=4 NGT, n=7 IGT and n=4 T2D for pooled islet gene expression analysis.

**Case ID**	**RNA Integrity Number (RIN) [pooled islets]**	**Histological PI-INS in-Situ analysis**	***Pooled* islet genes expression analysis**	***Individual* islet genes expression analysis**
NGT 1	6.3	YES	YES	YES
NGT 2	1	YES	NO	NO
NGT 3	7.1	YES	YES	YES
NGT 4	8	YES	YES	NO
NGT 5	5.6	YES	YES	YES
IGT 1	7.4	YES	YES	YES
IGT 2	1	YES	NO	NO
IGT 3	6	YES	YES	NO
IGT 4	6.6	YES	YES	YES
IGT 5	5.5	YES	YES	NO
IGT 6	7.1	YES	YES	YES
IGT 7	5.1	YES	YES	NO
IGT 8	5.1	YES	YES	NO
IGT 9	1	YES	NO	NO
T2D 1	5.1	YES	YES	NO
T2D 2	6.9	YES	YES	YES
T2D 3	7	YES	YES	YES
T2D 4	5.1	YES	YES	YES

ESM Table 3. Quality control check on microdissected individual islet using RT-Real-Time PCR. Raw Ct values of the exocrine marker *AMY2A* gene expression in comparison with islet-specific *INS* gene expression. Data are presented for each analysed individual islet included in the study (obtained from n=3 NGT, n=3 IGT and n=3 T2D).

	NGT subjects		
	**Islet code**	**AMY2A (raw Ct)**	**INS (raw Ct)**
**NGT-1**	1a	40,0	13,6
	2a	37,8	13,7
	3a	40,0	14,4
	4a	37,5	14,2
	5a	32,7	14,4
	6a	34,1	16,3
	7a	36,6	15,0
	8a	35,5	13,6
	9a	40,0	15,9
	10a	37,0	16,2
**NGT-2**	1a	35,3	13,8
	2a	37,2	16,2
	3a	36,5	12,8
	4a	38,1	14,6
	5a	36,2	15,9
	6a	35,9	14,8
	1b	40,0	16,1
	2b	40,0	19,2
	3b	36,9	14,9
	4b	36,4	16,8
	5b	40,0	19,9
	6b	40,0	17,2
**NGT-3**	1a	40,0	15,3
	2a	40,0	15,2
	3a	40,0	16,3
	4a	40,0	18,0
	5a	40,0	14,1
	6a	40,0	16,3
	7a	40,0	15,1
	8a	31,6	15,8
	9a	40,0	13,6
	10a	37,7	15,4

	**IGT donors**		
	**Islet code**	**AMY2A**	**INS**
**I**	11a	34,3	12,6

	12a	40,0	12,6
	13a	35,0	15,2
	14a	36,2	16,6
	15a	34,8	16,6
	17a	36,2	13,7
	18a	34,3	18,2
**IGT-2**	7a	36,6	19,0
	8a	38,0	14,1
	9a	37,2	14,8
	10a	34,0	13,8
	11a	37,9	16,8
	12a	33,5	14,5
	7b	40,0	14,9
	8b	34,1	16,8
	9b	32,6	16,3
	10b	40,0	14,7
	11b	38,8	15,2
	12b	33,5	15,9
**IGT-3**	11a	35,9	13,6
	12a	29,2	15,1
	13a	37,9	15,7
	14a	32,9	13,7
	15a	34,0	14,4
	16a	40,0	15,4
	17a	29,9	17,0
	18a	34,7	15,9
	19a	30,8	15,5
	20a	36,3	16,1
	21a	31,3	13,3
	**T2D donors**		
	**Islet code**	**AMY2A**	**INS**
**T2D-1**	19a	33,2	15,7
	20a	40,0	19,6
	21a	34,5	16,0
	22a	40,0	13,9
	23a	32,5	18,5
	24a	40,0	16,3
	25a	36,0	14,9
	26a	36,5	14,7
	27a	35,9	15,7
	28a	34,3	15,4
**T2**	13a	40,0	14,6
	14a	37,5	14,7

	15a	35,3	14,6
	17a	30,6	16,7
	13b	32,4	22,0
	14b	40,0	15,3
	15b	40,0	15,6
	16b	38,2	15,3
	17b	36,8	13,8
	18b	40,0	16,5
**T2D-3**	22a	40,0	14,2
	23a	36,9	17,5
	24a	32,4	15,3
	25a	33,0	14,7
	26a	36,8	17,3
	27a	36,3	15,6
	28a	30,4	13,5

ESM Table 4. List of selected genes reporting Official ID and gene names alongside their main biological processes.

Official ID	Gene name	Main biological process
INS	Insulin	Beta-cell function
GCG	Glucagon	Alpha cell function
PDX1	Pancreas/duodenum homeobox protein 1	Beta-cell differentiation
FOXA2	Forkhead box protein A2	Beta-cell differentiation
UCN3	Urocortin-3	Beta-cell differentiation
FOXO1	Forkhead box protein O1	Beta-cell phenotype
MAFA	V-maf musculoaponeurotic fibrosarcoma oncogene homolog A	Beta-cell phenotype
NKX6.1	Homeobox protein NK-6 homolog A	Beta-cell phenotype
PCSK1	Prohormone convertase 1	Proinsulin processing
PCSK2	Prohormone convertase 2	Proinsulin processing
CPE	Carboxypeptidase E	Proinsulin processing
CHGB	Chromogranin B	Proinsulin processing
GCK	Glucokinase	Beta-cell function
SLC2A2	Solute Carrier Family 2 Member 2	Beta-cell function
ACLY	ATP citrate lyase	Beta-cell function
IAPP	Islet amyloid polypeptide	Beta-cell function
ABCC8	ATP Binding Cassette Subfamily C Member 8	Beta-cell function
S100A9	S100 Calcium-Binding Protein A9	Inflammation
CHGA	Chromogranin-A	Endocrine marker
CRTC1	CREB-regulated transcription coactivator 1	Beta-cell proliferation
ALDH1A3	Aldehyde dehydrogenase family 1 member A3	Beta-cell dedifferentiation
VIMENTIN	Vimentin	Beta-cell dedifferentiation
CDH1	Cadherin-1	Cell adhesion
CADM1	Cell adhesion molecule 1	Cell adhesion
LMNB1	Laminin B1	Extracellular matrix
GRP78	Glucose-regulated protein	Adaptive-UPR
XBP1	Splicing of X-box binding protein 1	Adaptive-UPR
PDIA1	Protein disulfide isomerase	Adaptive-UPR
ATF6	Activating transcription factor 6	Adaptive-UPR
ERP27	Endoplasmic reticulum resident 27	ER stress and folding
ERO1B	ER oxidoreductin 1β	ER stress and folding

RO60	RNA binding protein	ER stress and folding
CCT4	Chaperonin containing TCP1 subunit 4	ER stress and folding
PERK	Pancreatic ER kinase	Adaptive and terminal UPR
IRE1α	Inositol-requiring enzyme 1α	Adaptive and terminal UPR
CHOP	C/EBP homologous protein	Terminal-UPR
SEL1L	Protein sel-1 homolog 1	ER-associated degradation
HRD1	ERAD-associated E3 ubiquitin-protein ligase HRD1	ER-associated degradation
AMY2A	Amylase alpha 2A (pancreatic)	Exocrine-specific marker

ESM FIGURES

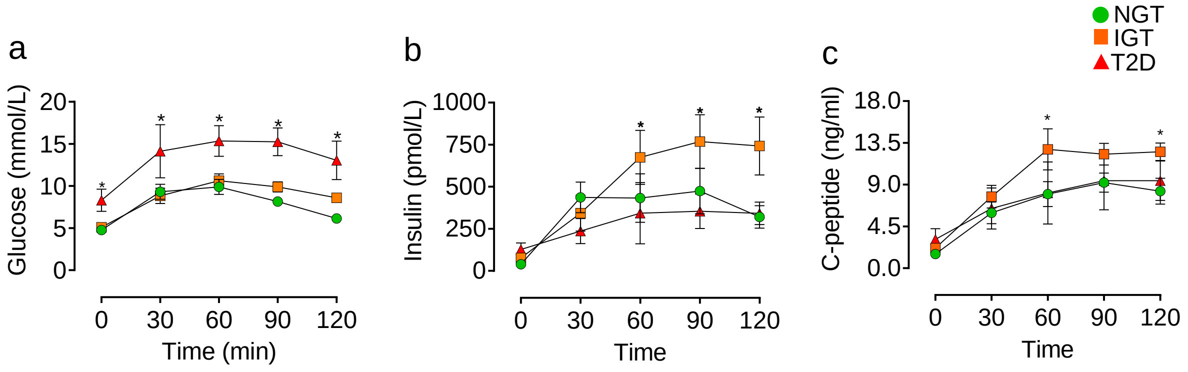

ESM Figure 1. (**a**) Glucose, (**b**) insulin, (**c**) C-peptide levels during OGTT before partial pancreatectomy in NGT (green circle), IGT (orange square) and T2D (red diamond). *$p \leq 0.05$, was considered statistically significant for glucose, insulin and C-peptide levels for each time point**.**

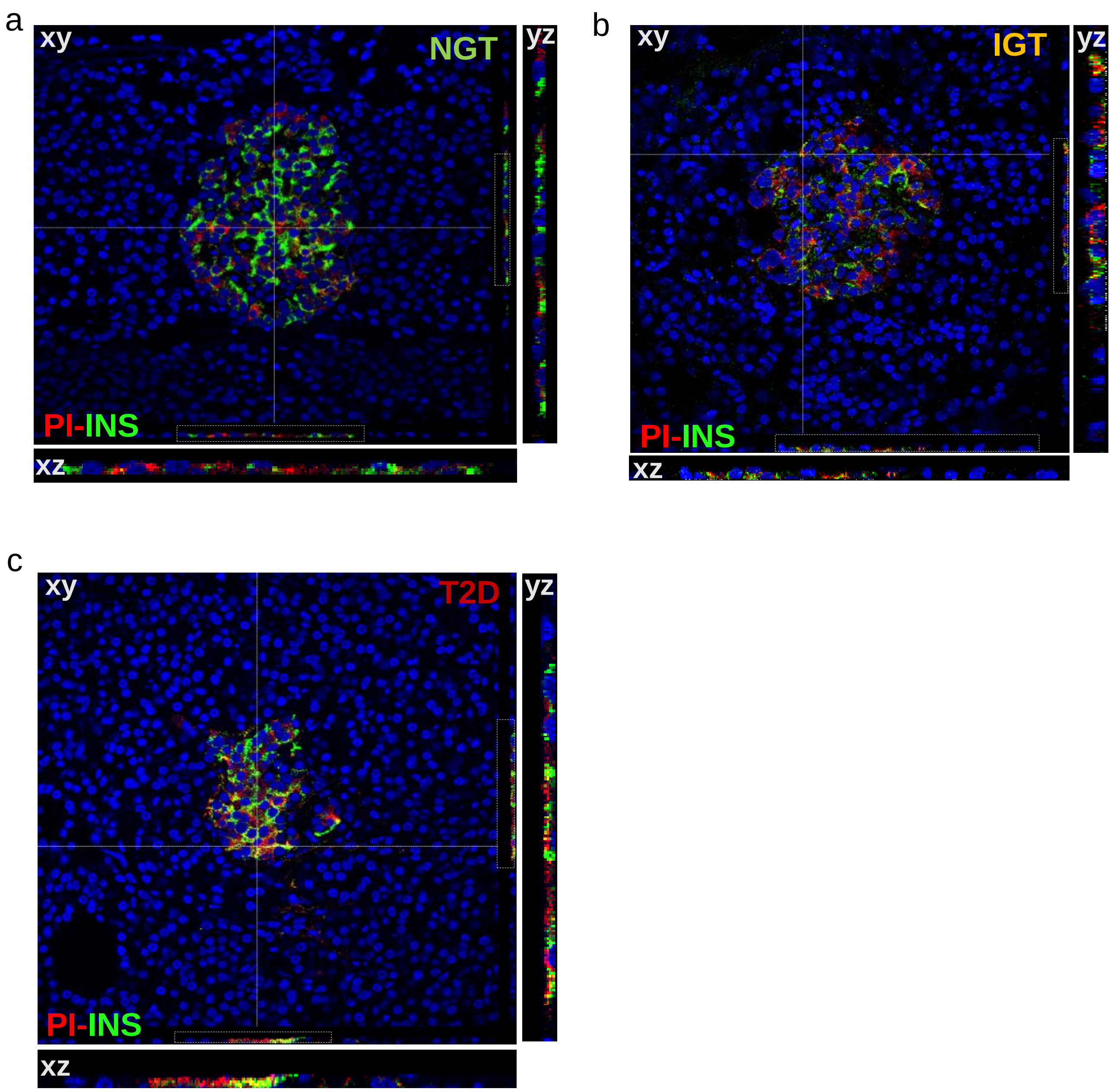

ESM Figure 2. (**a-c)** Representative z-stack 2D projection analysis of PI-INS immunofluorescence staining of human pancreatic tissue sections of NGT, IGT and T2D living donors. Images show the z-stack 2D projection (xy, xz, yz) of serial confocal planes (n=10) relative to the proinsulin (PI) and insulin (INS) immunofluorescence staining of pancreatic tissue sections of NGT (**a**), IGT (**b**) and T2D (**c**). Crossair in xy images shows the location of xz and yz projections. Zoom-in of xz and yz projections are related to the white dotted rectangle in the main figure.

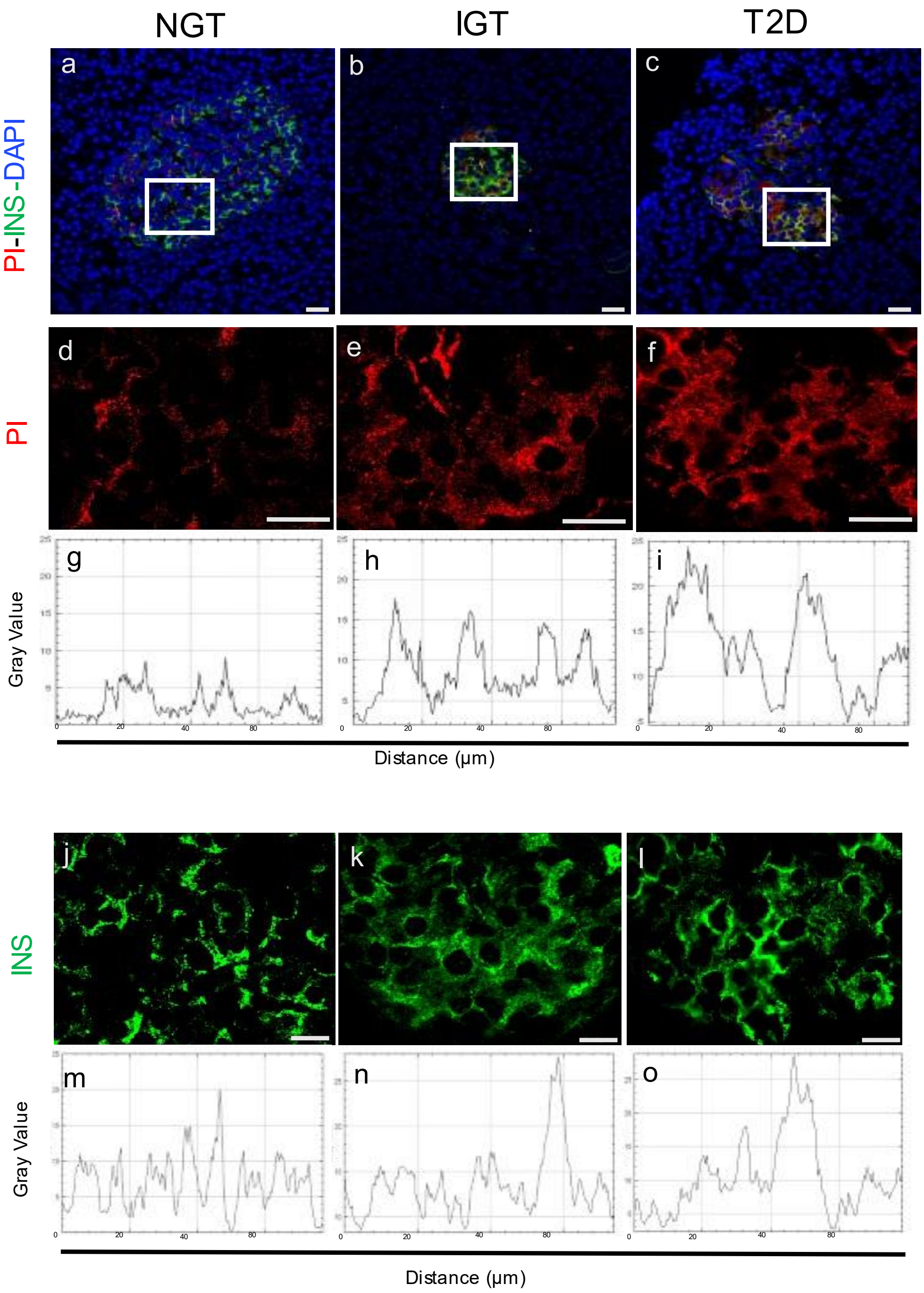

ESM Figure 3. Pixel intensity distribution analysis of PI and INS in representative pancreatic islets of NGT, IGT and T2D living donors. PI is increased while insulin remains unchanged from NGT to IGT to T2D. Panels a-c: images of islets stained for proinsulin (PI, red), insulin (INS, green), nuclei (blue) in NGT (panel a), IGT (panel b) and T2D (panel c) pancreatic tissues. White squares depict the zoom-in part of the image shown in panels d-f and in panels j-l. Panels d-f: representative zoom-in images of proinsulin staining (red) of NGT, IGT and T2D pancreatic sections shown in panels a-c. Panels g-i: two dimensional graphs reporting PI pixel intensities (gray scale values) across images of NGT (panel g), IGT (panel h) and T2D (panel i). Panels j-l: representative zoom-in images of insulin staining (green) of NGT, IGT and T2D pancreatic sections shown in panels a-c. Panels m-o: two dimensional graphs reporting insulin pixel intensities (gray scale values) across images of NGT (panel g), IGT (panel h) and T2D (panel i). Distances are shown in µm. Scale bars in panels a-c, in panels d-f and j-l are 20 µm.

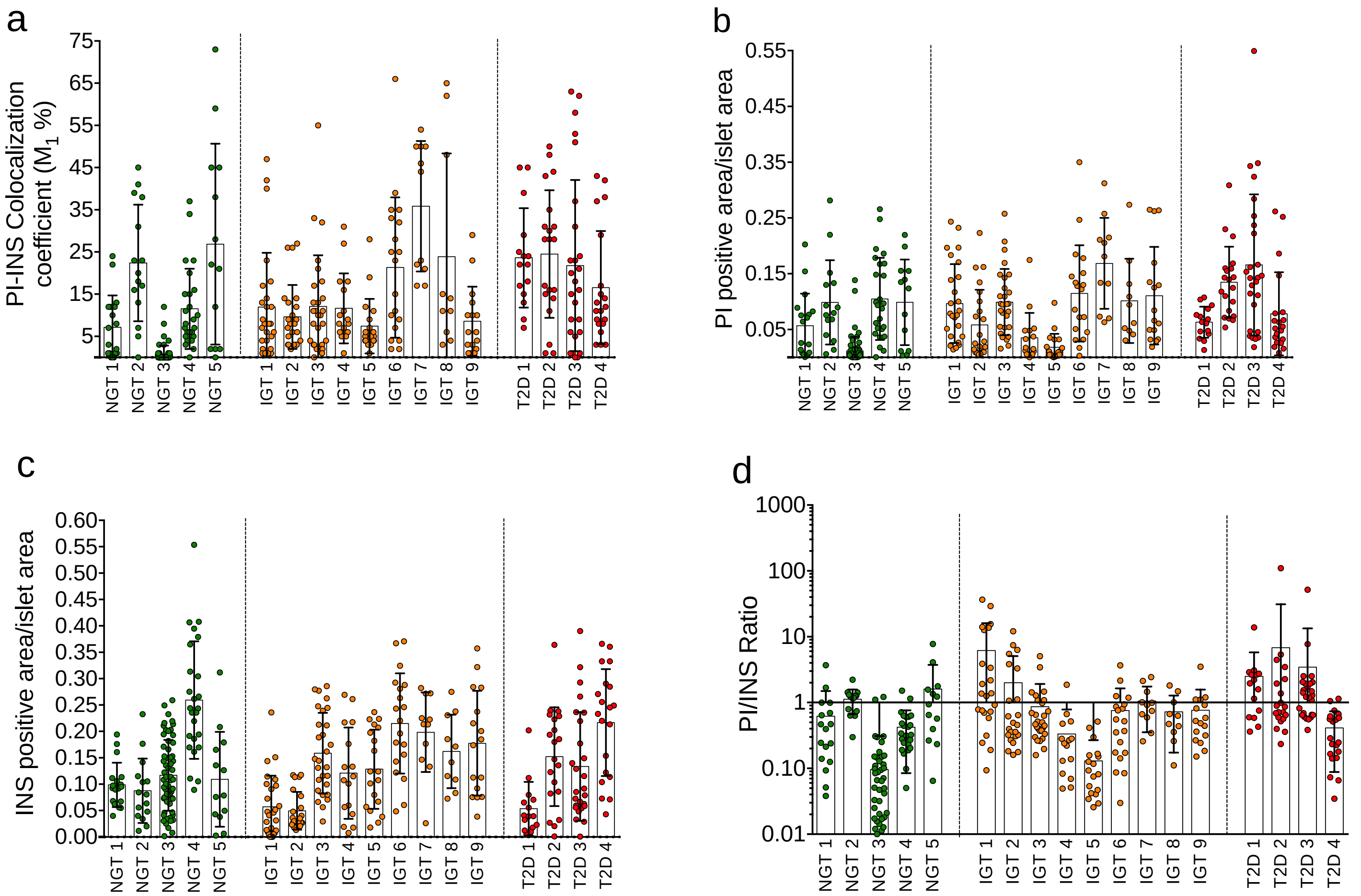

ESM Figure 4. Analysis of PI-INS immunofluorescence staining in pancreatic tissue sections of individual NGT (n=5), IGT(n=9) and T2D subjects (n=4). Bar plots showing PI positive area (**a**), INS positive area (**b**), PI-INS area ratio (**c**), PI-INS colocalization coefficient (**d**), of all cases analysed. Each dot represents an individual islet. Mean ± S.E. values are reported for each case.

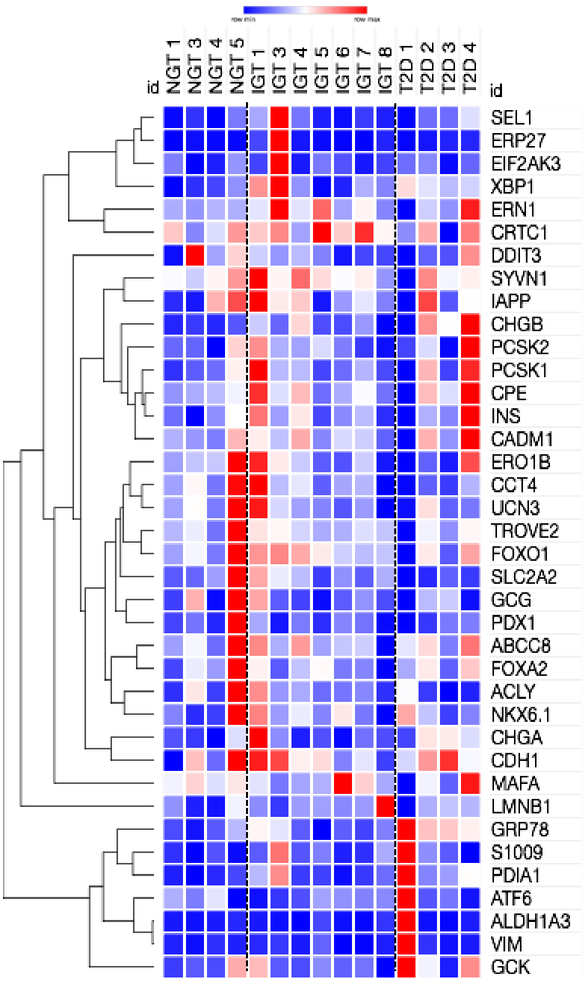

ESM Figure 5. Heatmap reporting the expression analysis of a selected list of genes involved in the ER stress and beta-cell function and phenotype in LCM-pancreatic islets from NGT, IGT and T2D living donors.

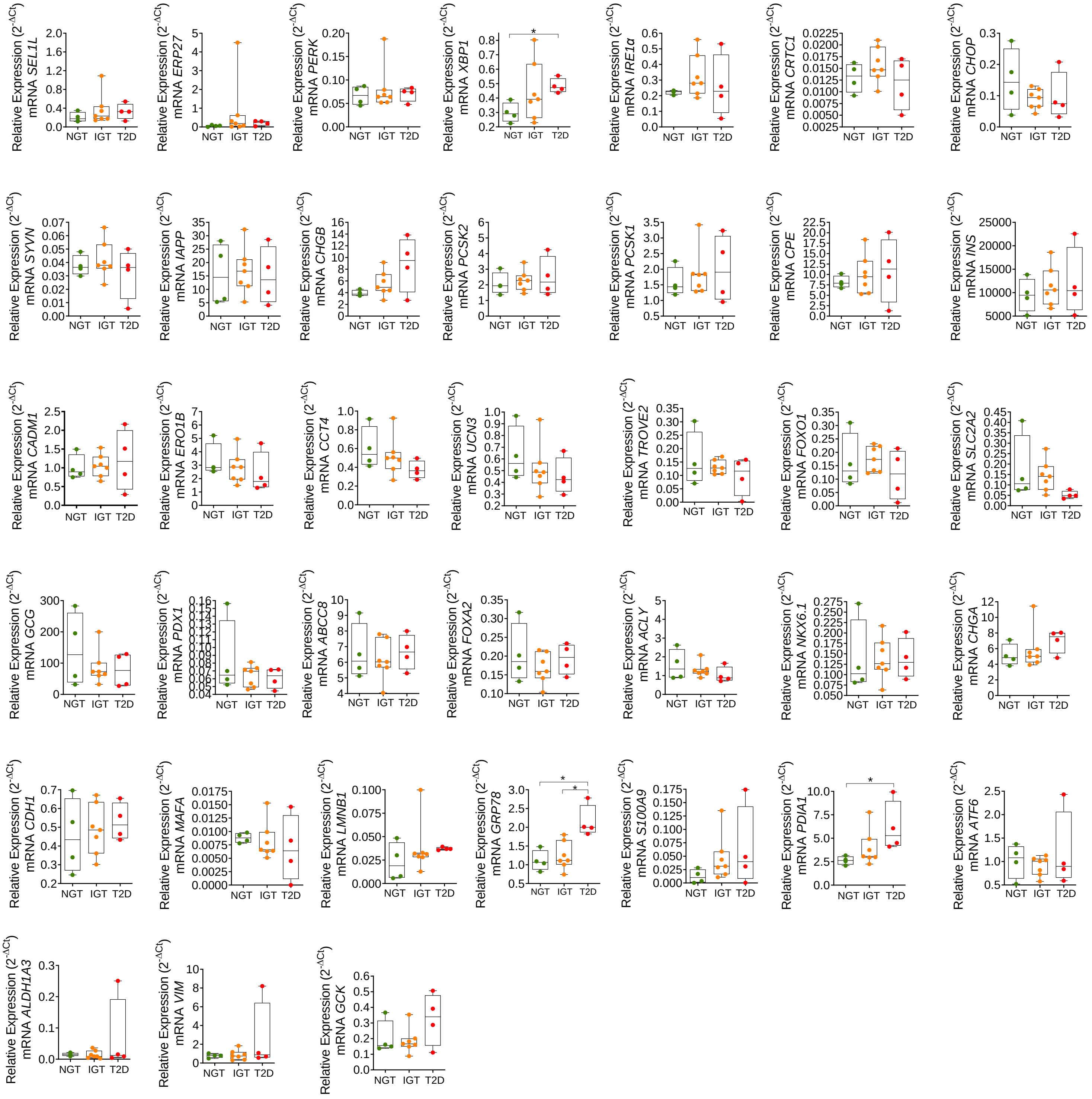

ESM Figure 6. Real-Time PCR gene expression analysis of pooled LCM pancreatic islets of NGT, IGT and T2D subjects reported in the hierarchical clustering analysis of ESM Figure 5. Box plot graphs show the expression of 38 genes associated with beta-cell function, ER stress/UPR pathways in microdissected pancreatic islets of n=4 NGT, n=7 IGT and n=4 T2D. Data are reported as 2 $^{-dcT}$ values. Mean± S.D. values are reported. **p*<0.05, ***p*<0.01, ****p*<0.001, One-way ANOVA and Tukey's multiple comparisons post-test.

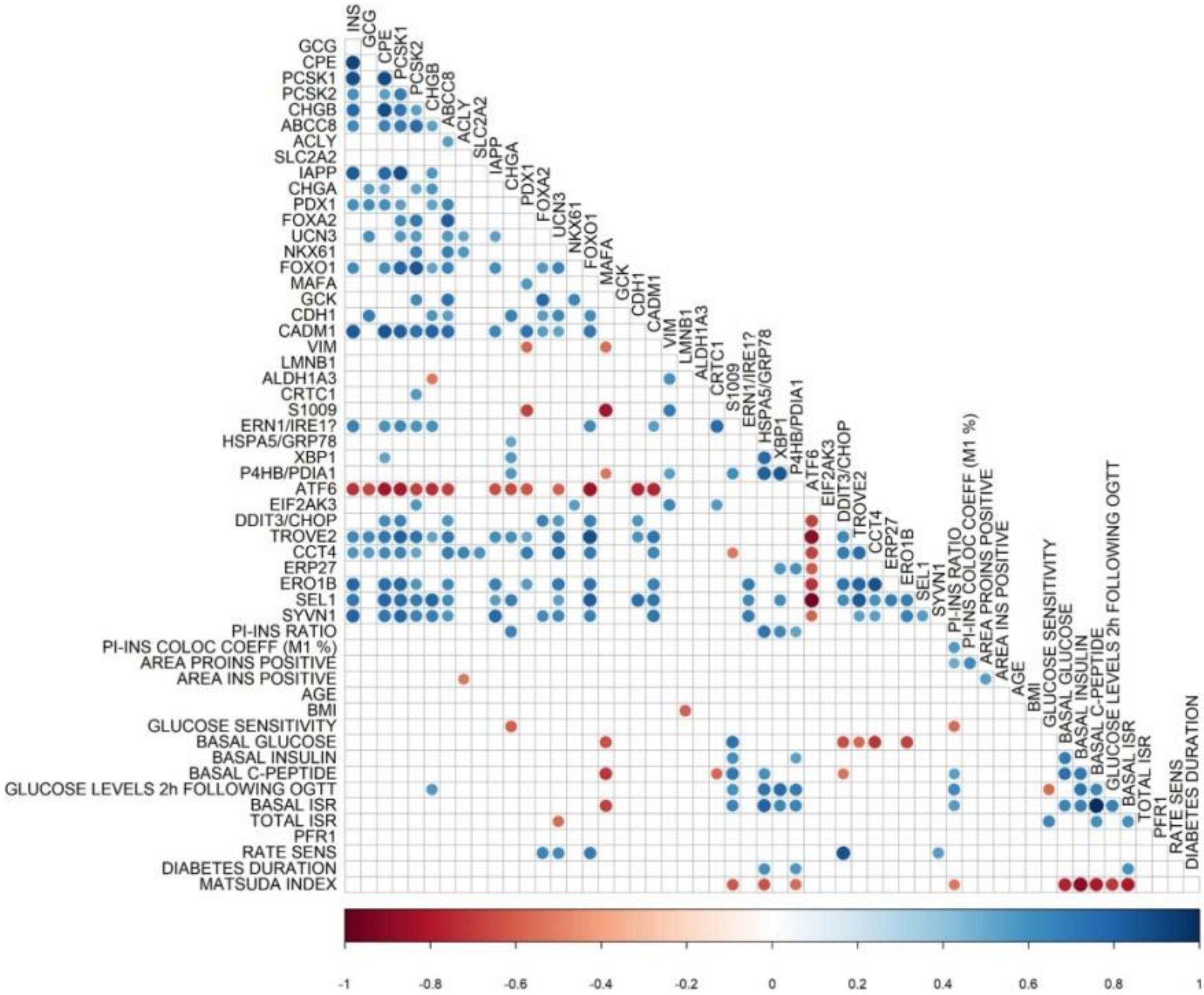

ESM Figure 7. Correlation matrix showing the *r*-values [scale colours from red (-1) to blue (1)] of significant correlations between gene expression levels, in situ PI-INS imaging parameters and clinical outcomes in NGT, IGT and T2D living donors. Correlations were performed using the Spearman R-test, taking into account $p \leq 0.05$. The statistical significance of the Spearman *r* test is indicated with the presence and size of coloured dots.

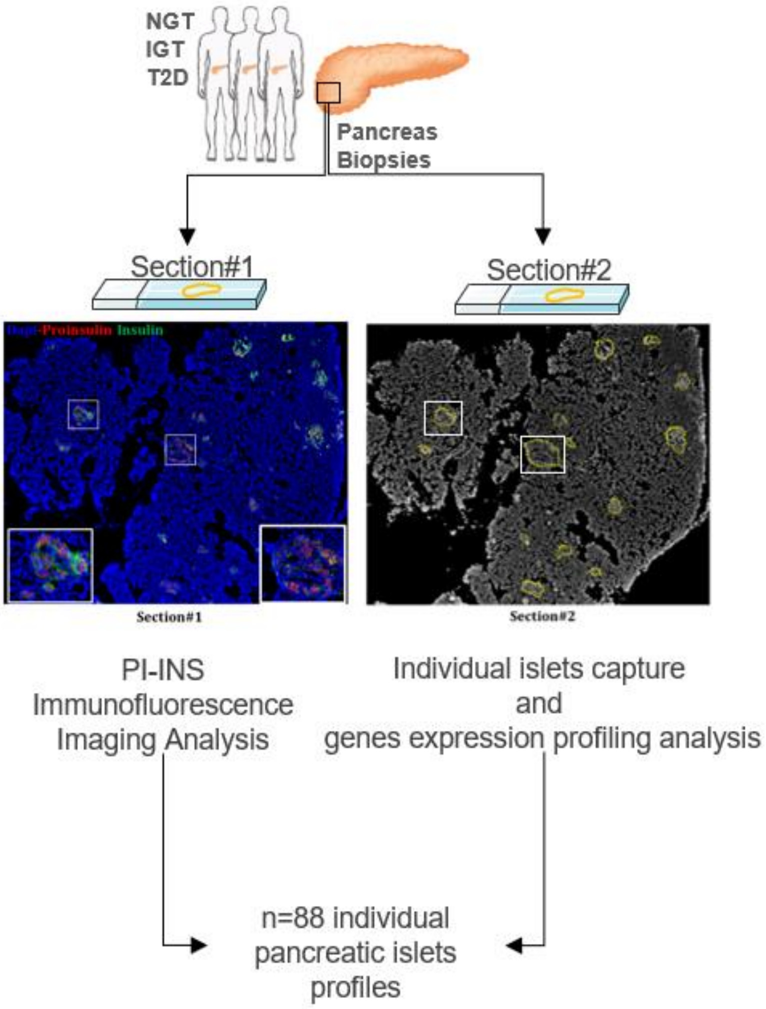

ESM Figure 8. Individual human islet phenotyping reveals a direct association between proinsulin processing, ER stress and loss of beta-cell identity during in-vivo metabolic alterations. Workflow diagram of individual islet phenotype from consecutive serial pancreatic sections of NGT, IGT and T2D patients. A total of 88 different individual pancreatic islets were profiled by molecular and immunofluorescence analysis.

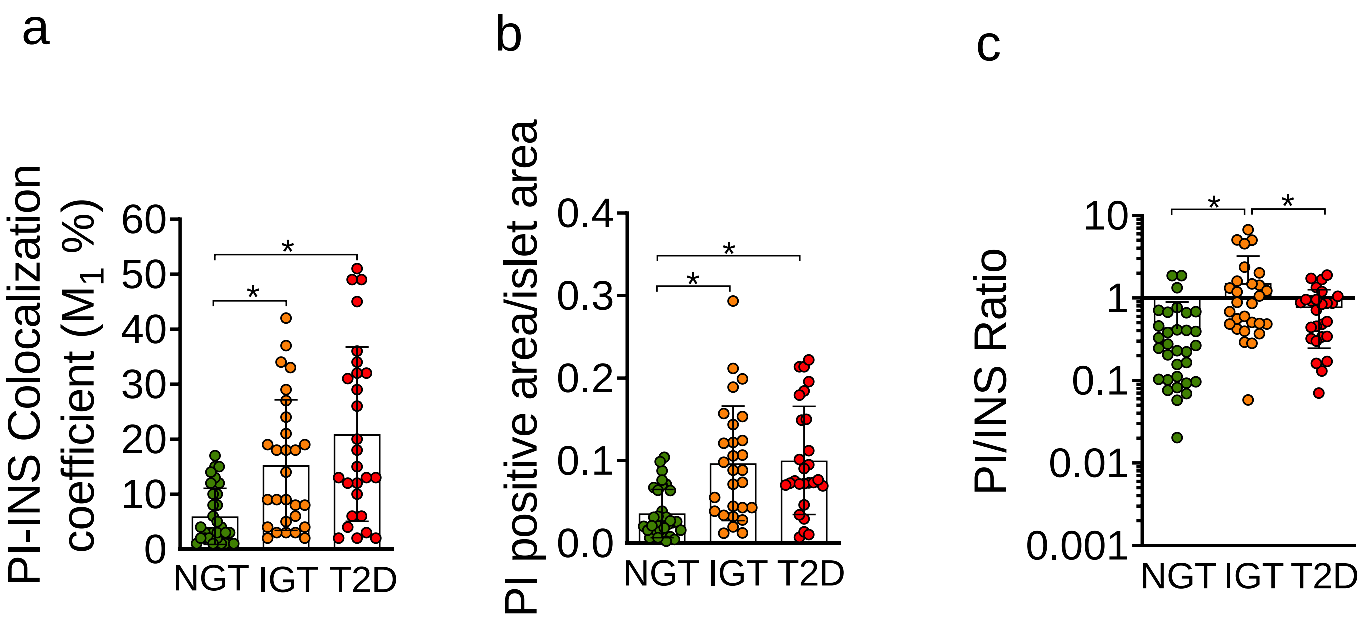

ESM Figure 9. Individual pancreatic islets PI-INS immunofluorescence analysis parameters. (**a-c**) Bar graphs showing immunofluorescence analysis parameters: PI-INS colocalization coefficient M_1 (**a**), PI positive area/islet area (**b**), and the PI-INS area ratio (**c**), in individual mapped pancreatic islets from n=3 NGT, n=3 IGT and n=3 T2D patients. *p<0.05, **p<0.01, ***p<0.001, One-way ANOVA and Tukey's multiple comparisons post-test. Data are presented as individual values for each pancreatic islet alongside mean ± S.D.

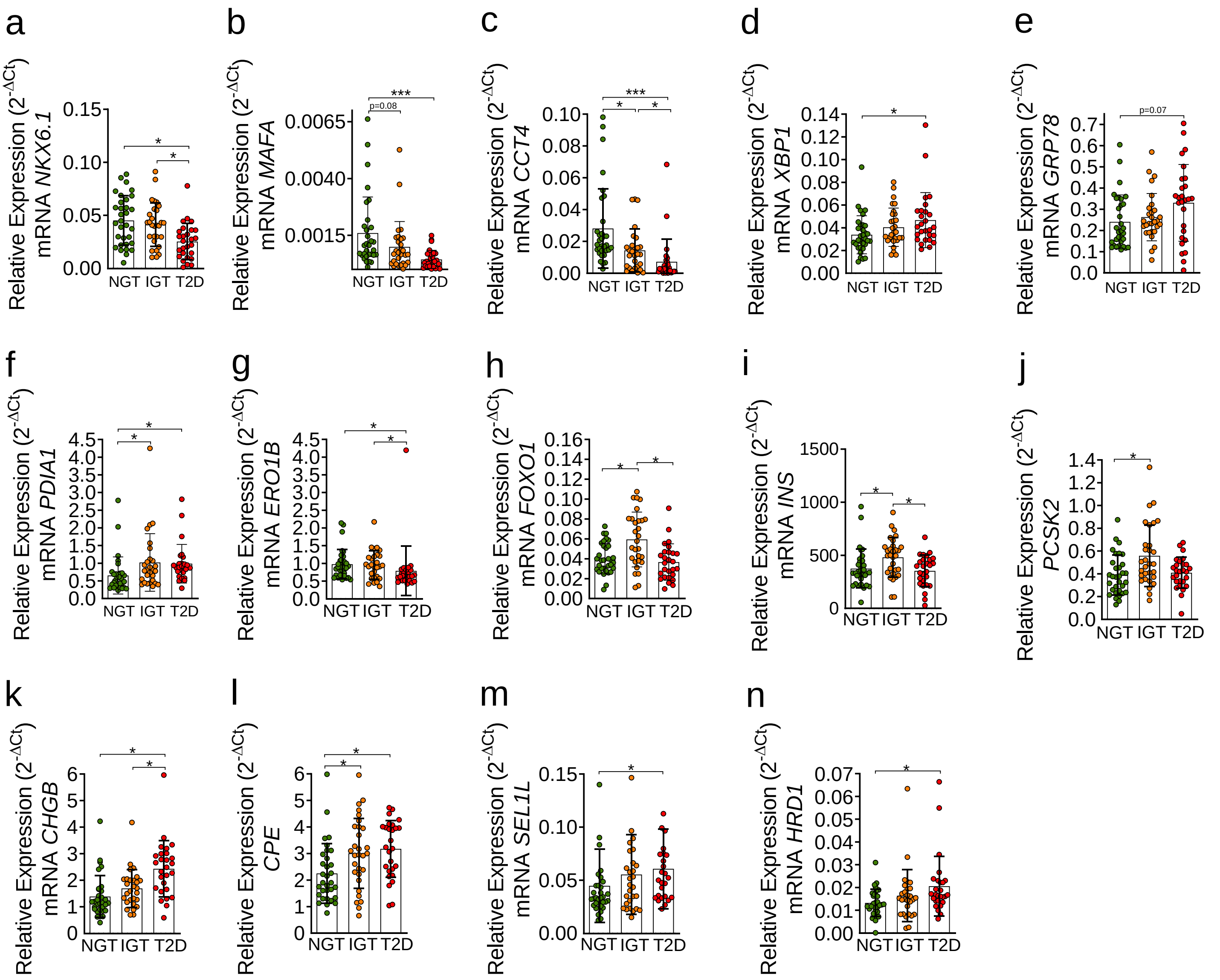

ESM Figure 10. (A-N) Bar charts showing the expression of *NKX6.1* (**a**), *MAFA* (**b**), *CCT4* (**c**), *XBP1* (**d**), *GRP78* (**e**), *PDIA1* (**f**), *ERO1B* (**g**), *FOXO1* (**h**), *INS* (**i**), *PCSK2* (**j**), *CHGB* (**k**), *CPE* (**l**), *SEL1L* (**m**) and *HRD1* (**n**), evaluated through RT-Real-Time PCR in n=32 individual NGT, n=29 IGT and n=28 individual islets of T2D subjects isolated with LCM. *p<0.05, **p<0.01, ***p<0.001, Kruskal Wallis with Dunn's multiple comparison test.

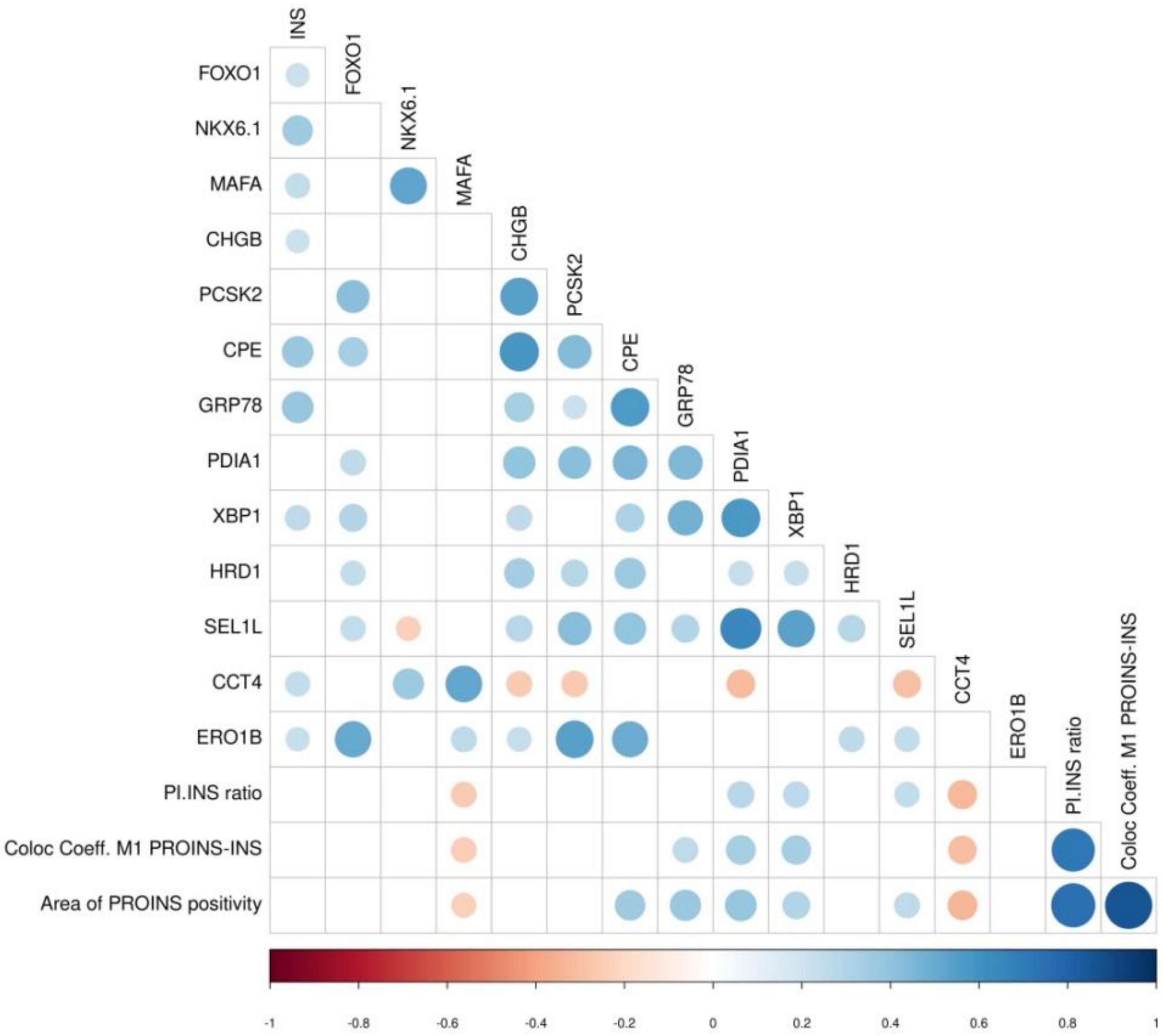

ESM Figure 11. Correlation matrix showing correlation coefficients among individual islet gene expression values and in situ immunofluorescence parameters associated with in situ proinsulin and insulin staining parameters. Colour key indicates the positive (dark blue) and negative (dark red) correlation *r*-values. The statistical significance of the Spearman r test ($p \leq 0.05$) is indicated with the presence and size of blue or red dots.

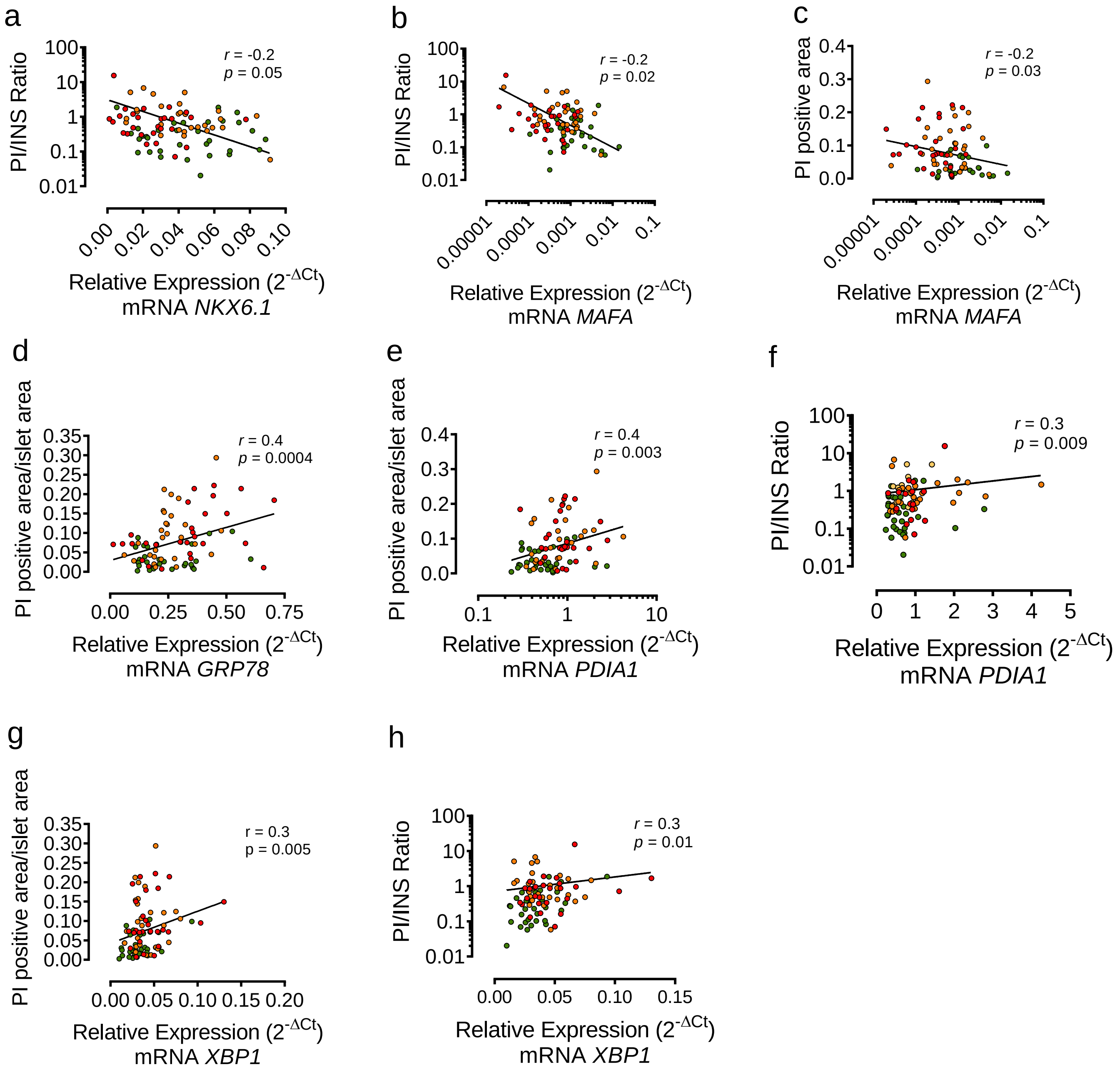

ESM Figure 12. Selection of scatter plots of correlation analysis shown in in ESM Figure 10. Correlation among genes expression and PI and INS in situ imaging parameters measured at individual islet levels are shown. *p*-values and *r*-values were obtained using Spearman r test taking into account P < 0.05.
